# *Vps33b* is crucial for structural and functional hepatocyte polarity

**DOI:** 10.1016/j.jhep.2017.01.001

**Published:** 2017-05

**Authors:** Joanna Hanley, Dipok Kumar Dhar, Francesca Mazzacuva, Rebeca Fiadeiro, Jemima J. Burden, Anne-Marie Lyne, Holly Smith, Anna Straatman-Iwanowska, Blerida Banushi, Alex Virasami, Kevin Mills, Frédéric P. Lemaigre, A.S. Knisely, Steven Howe, Neil Sebire, Simon N. Waddington, Coen C. Paulusma, Peter Clayton, Paul Gissen

**Affiliations:** 1 UCL Institute of Child Health, University College London, London WC1N 1EH, UK; 2 Organ Transplantation Centre and Comparative Medicine Department, King Faisal Specialist Hospital and Research Centre, Riyadh 11211, Saudi Arabia; 3 MRC Laboratory for Molecular Cell Biology, University College London, London WC1E 6BT, UK; 4 UCL Department of Statistical Science, University College London, London WC1E 6BT, UK; 5 Histopathology Department, Camelia Botnar Laboratories, Great Ormond Street Hospital for Children NHS Trust, London WC1N 3JH, UK; 6 Université Catholique de Louvain, de Duve Institute, 1200 Brussels, Belgium; 7 Institut für Pathologie, Medizinische Universität Graz, 8036 Graz, Austria; 8 UCL Institute for Women’s Health, University College London, London WC1E 6AU, UK; 9 Antiviral Gene Therapy Research Unit, Faculty of Health Sciences, University of the Witswatersrand, Johannesburg 2193, South Africa; 10 Tytgat Institute for Liver and Intestinal Research, Academic Medical Center, 1105 BK Amsterdam, Netherlands; 11 Inherited Metabolic Disease Unit, Great Ormond Street Hospital for Children NHS Trust, London WC1N 3JH, UK

**Keywords:** Vps33b, Hepatocyte polarity, Canalicular membrane, Cholestasis, Protein trafficking, Rab11a, Gene transfer, Bile canaliculi, Bile, Liver diseases

## Abstract

**Background & Aims:**

In the normal liver, hepatocytes form a uniquely polarised cell layer that enables movement of solutes from sinusoidal blood to canalicular bile. Whilst several cholestatic liver diseases with defects of hepatocyte polarity have been identified, the molecular mechanisms of pathogenesis are not well defined. One example is arthrogryposis, renal dysfunction and cholestasis syndrome, which in most patients is caused by *VPS33B* mutations. VPS33B is a protein involved in membrane trafficking that interacts with RAB11A at recycling endosomes. To understand the pathways that regulate hepatocyte polarity better, we investigated VPS33B deficiency using a novel mouse model with a liver-specific *Vps33b* deletion.

**Methods:**

To assess functional polarity, plasma and bile samples were collected from *Vps33b* liver knockout (*Vps33b*^fl/fl^*-AlfpCre*) and control (*Vps33b*^fl/fl^) mice; bile components or injected substrates were quantitated by mass spectrometry or fluorometry. For structural analysis, livers underwent light and transmission electron microscopy. Apical membrane and tight junction protein localisation was assessed by immunostaining. Adeno-associated virus vectors were used for *in vivo* gene rescue experiments.

**Results:**

Like patients, *Vps33b*^fl/fl^-AlfpCre mice showed mislocalisation of ATP-binding cassette proteins that are specifically trafficked to the apical membrane via Rab11a-positive recycling endosomes. This was associated with retention of bile components in blood. Loss of functional tight junction integrity and depletion of apical microvilli were seen in knockout animals. Gene transfer partially rescued these defects.

**Conclusions:**

*Vps33b* has a key role in establishing structural and functional aspects of hepatocyte polarity and may be a target for gene replacement therapy.

**Lay summary:**

Hepatocytes are liver cells with tops and bottoms; that is, they are polarised. At their bottoms they absorb substances from blood. They then, at their tops, secrete these substances and their metabolites into bile. When polarity is lost, this directional flow of substances from blood to bile is disrupted and liver disease follows. In this study, using a new mouse model with a liver-specific mutation of *Vps33b,* the mouse version of a gene that is mutated in most patients with arthrogryposis, renal dysfunction and cholestasis (ARC) syndrome, we investigated how the *Vps33b* gene product contributes to establishing hepatocyte polarity. We identified in these mice abnormalities similar to those in children with ARC syndrome. Gene transfer could partly reverse the mouse abnormalities. Our work contributes to the understanding of *VPS33B* disease and hepatocyte polarity in general, and may point towards gene transfer mediated treatment of ARC liver disease.

## Graphical abstract

**Figure:**
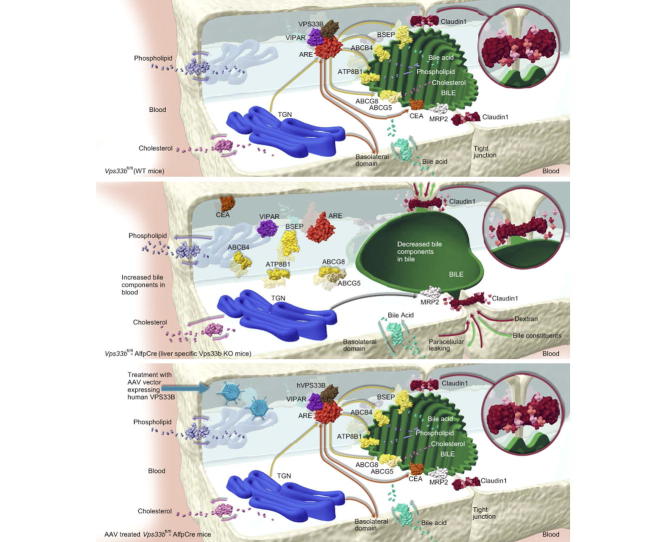

## Introduction

In metazoans, the development of three-dimensional body structures depends on the generation of polarised epithelial layers. Polarisation of epithelial cells is a complex process that requires the cooperation of multiple factors including cell junction formation, extracellular matrix interactions and intracellular protein trafficking [1]. Correct interaction of these factors enables the establishment of discrete apical and basolateral membrane domains. This then promotes normal epithelial cell function by allowing directional solute absorption and secretion [2].

In hepatocytes, the basolateral membrane faces sinusoidal blood and one or more apical domains contribute to the formation of bile canaliculi in conjunction with adjacent hepatocytes. This polarisation enables the directional flow of molecules from sinusoidal blood to canalicular bile and, as such, is crucial for normal biliary physiology [3]. Genetic disorders of hepatic tight junction formation and protein trafficking entail a loss of hepatocyte polarity. This impaired hepatocyte polarity causes pathophysiological features similar to those of cholestatic liver diseases caused by mutations in genes encoding apical ATP-binding cassette transporters [4], [5], [6], [7].

One such polarity-loss disorder is arthrogryposis renal dysfunction and cholestasis syndrome (ARC). ARC is an autosomal recessive multisystem disorder. It has a spectrum of severity and typically is characterised by congenital joint contractures, renal tubular acidosis and neonatal jaundice leading to death in infancy [8]. However, in milder cases patients survive into adulthood and have raised serum bile acid values but normal bilirubin levels [9]. Approximately 75% of ARC patients harbour germline mutations in *VPS33B*. VPS33B is orthologous to the yeast Sec-1-Munc18 family protein Vps33p: a class C vacuolar protein sorting (vps) protein that regulates SNARE-mediated vesicle fusion [10]. Class C vps proteins are core constituents of the homotypic fusion and vacuole protein sorting (HOPS) and class C core vacuole/endosome tethering (CORVET) multiprotein complexes, crucial in several stages of vesicular trafficking [11]. Whilst the Vps33p ortholog VPS33A is recognised as a member of the mammalian HOPS complex [12], the function of VPS33B is associated with the activity of Rab11 family members [13].

Rab11a is involved in protein trafficking via the apical recycling endosome (ARE) and, thereby, biogenesis of the apical membrane domain [14]. Canalicular proteins that are specifically trafficked via Rab11a-positive ARE are mislocalised in ARC patient liver [8], [11]. Whilst patients’ serum aminotransferase and gamma-glutamyl transpeptidase (γGT) activities are normal, serum alkaline phosphatase (ALP) activity is substantially elevated [8].

To treat the human liver diseases associated with failure to regulate apical-basolateral polarity, one must understand the pathways that govern hepatocyte polarisation. We therefore investigated the molecular pathology of ARC liver disease to elucidate the role of VPS33B in establishing hepatocyte polarity in mammals. We generated and characterised a novel murine model with a liver-specific *Vps33b* gene mutation (*Vps33b*^fl/fl^*-AlfpCre*) that recapitulates key aspects of ARC liver disease. In *Vps33b*^fl/fl^-AlfpCre animals we observed structural and functional defects of hepatocyte polarity associated with failure to generate the canalicular membrane correctly. We could, in part, reverse these defects by adeno-associated virus mediated gene transfer.

## Materials and methods

### *Vps33b*^fl/fl^-AlfpCre mouse generation

Mice with *Vps33b* exons 2–3 flanked by loxP sites were generated by Artemis Pharmaceuticals (Cologne, Germany) as described and crossed onto a C57BL/6 J background [15]. Mice were further crossed with AlfpCre-recombinase expressing animals to obtain *Vps33b*^fl/fl^-AlfpCre mice with hepatocyte and cholangiocyte specific *Vps33b* deletion [16]. Animals were sacrificed at 14–16 weeks of age and Cre-negative, age matched littermates were used as wild-type controls. For experiments on a cholic acid (CA) diet, 8 week old mice were fed 0.5% CA supplemented chow (TestDiet® Europe, London, UK) for 6 weeks and sacrificed immediately thereafter at age 14 weeks. For histologic assessment in older animals, mice were aged for 10 to 12 months and fed a normal chow diet. All experimental animal groups were of mixed gender. All procedures were undertaken with United Kingdom Home Office approval in accordance with the Animals (Scientific Procedures) Act of 1986.

### Mass spectrometry

Bile acids were quantified by UPLC-MS/MS [17]. Bile acid concentrations in bile were corrected by flow rate. Details are provided in the Supplementary methods section.

### Transmission electron microscopy (TEM)

Details of TEM procedures [13] are provided in the Supplementary methods section.

### Biliary secretion analysis

For analysis of bile composition, bile was collected by cannulating the gall bladder of 14–16-week-old CA fed mice using polyethylene (PE-10) tubing following midline laparotomy and ligation of the common bile duct. Analysis of biliary cholyl-L-lysyl-fluorescein (CLF) secretion via MRP2 was carried out using *in vivo* methods [18]. Integrity of hepatic tight junction barrier function was measured using 40 kDa fluorescein isothiocyanate (FITC)-conjugated dextran (FD-40) [19]. Results obtained from CLF and FD-40 fluorometric analysis were corrected for bile flow rate.

### Microarray analysis

Total RNA was extracted from liver samples using the RNAeasy Microkit per manufacturer’s instructions (Qiagen, Manchester, UK). RNA transcript levels were measured using Affymetrix Mouse Gene 2.0 microarrays. Details of *in silico* gene expression and pathway analysis are described in the Supplemental methods section.

The data discussed in this publication have been deposited in NCBI’s Gene Expression Omnibus [20] and are accessible through GEO series accession number GSE83192 (https://www.ncbi.nlm.nih.gov/geo/query/acc.cgi?acc=GSE83192).

### Adeno-associated virus (AAV) vector production

All ssAAV2/8 vectors were made by the adenovirus-free transient transfection method, using a chimeric AAV2 Rep-8Cap packaging plasmid (pAAV2-8) and an adenoviral helper plasmid [21]. Vectors were purified [22] and vector genome (vg) titres were determined by standard alkaline gel based methods [23]. Details of molecular cloning procedures are provided in the Supplemental methods section.

### Serum and bile biochemical assays, immunoblotting and histological and immunostaining

All the above procedures are described in the Supplemental methods section.

## Results

### The ARC liver phenotype is recapitulated in *Vps33b*^fl/fl^-AlfpCre mice and exacerbated by CA feeding

Loss of *Vps33b* expression in hepatocytes of *Vps33b*^fl/fl^-AlfpCre animals was confirmed by fluorescence *in situ* hybridisation and Western blotting techniques (Supplementary Fig. 1A and B).

**Fig. 1:**
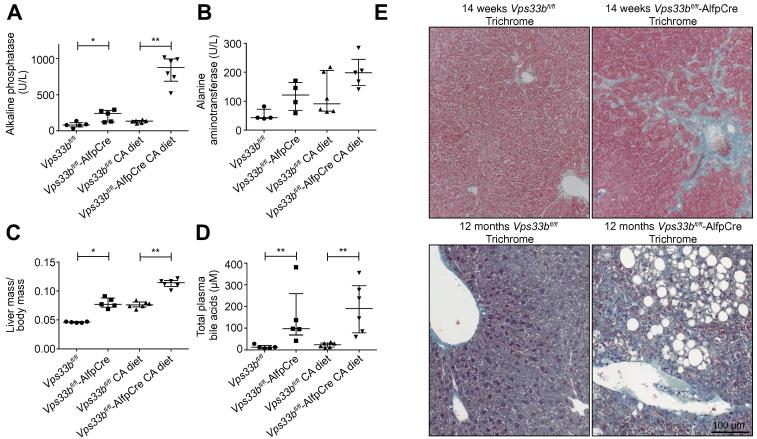
**ARC liver disease in *Vps33b*^fl/fl^-AlfpCre mice.** (A) Plasma ALP activity, (B) plasma ALT activity (C) liver mass and (D) total plasma bile acid levels assessed in 14-week-old normal chow fed and cholic acid (CA) fed *Vps33b*^fl/fl^-AlfpCre and *Vps33b*^fl/fl^ mice (n = 4–6). (E) Trichrome staining of liver sections from 14-week-old CA fed mice and 12-month-old mice fed with normal diet (n = 3). Graphs are presented with medians and IQR. *p* values (Mann Whitney *U* test): ALP activity, *p* = 0.0317 and *p* = 0.0022; ALT activity *p* = 0.0591 and *p* = 0.1775; plasma bile acids, *p* = 0.0043 and *p* = 0.0022; liver mass, *p* = 0.0117 and *p* = 0.0050 (normal chow fed and CA fed comparisons respectively).

To assess hepatobiliary injury associated with *Vps33b* mutation, levels of plasma ALP activity; alanine aminotransferase (ALT) activity and bilirubin concentrations were measured in 14-week-old animals fed normal chow or a 0.5% CA diet (n = 4–6). As in ARC patients, a significant increase in plasma ALP activity was detected in normal chow fed *Vps33b*^fl/fl^-AlfpCre mice (3-fold, median: 241.0 U/L, IQR: 123.5–283.5 U/L) in comparison to Cre-negative *Vps33b*^fl/fl^ littermates (median: 80.0 U/L, IQR: 54.0–113.50 U/L) (Fig.1A, *p* = 0.0317, Mann Whitney *U* test). In CA fed animals, a greater increase in ALP activity (6.5-fold) was observed in the plasma of *Vps33b*^fl/fl^-AlfpCre mice (median: 874.5 U/L, IQR: 688.0–994.5 U/L) than in that of *Vps33b*^fl/fl^ mice (median: 134.0 U/L, IQR: 108–144.0 U/L) (Fig.1A, *p* = 0.0022, Mann Whitney *U* test). Whilst ALT activity levels appeared slightly raised in *Vps33b*^fl/fl^-AlfpCre animals, no significant difference in plasma ALT activity was found between *Vps33b*^fl/fl^ and *Vps33b*^fl/fl^-AlfpCre mice under both diet conditions (Fig.1B, normal diet *p* = 0.0591, CA diet *p* = 0.1775, Mann Whitney *U* test). Although ALT activity levels increased in both *Vps33b*^fl/fl^ and *Vps33b*^fl/fl^-AlfpCre animals following CA feeding, this increase was not statistically significant (*Vps33b*^fl/fl^ *p* = 0.0691, *Vps33b*^fl/fl^-AlfpCre *p* = 0.1111). Addition of CA to the diet may therefore have a slight effect on liver injury in both wild-type and knockout animals. CA feeding consistently increased liver mass in *Vps33b*^fl/fl^ and *Vps33b*^fl/fl^-AlfpCre mice (1.5-fold, *Vps33b*^fl/fl^ *p* = 0.0078, *Vps33b*^fl/fl^-AlfpCre *p* = 0.0043) and liver mass was significantly increased between *Vps33b*^fl/fl^ and *Vps33b*^fl/fl^-AlfpCre mice under both diet conditions (normal diet 1.7-fold; *p* = 0.0117, CA diet 1.5-fold; *p* = 0.0050) (Fig.1C). Plasma bilirubin concentrations were below detection level (<2 μmol/L) in all experimental groups.

As high plasma bile acid levels characterise ARC cholestasis [8], we measured bile acids in mouse plasma by mass spectrometry (n = 5–6). Concordantly, median total bile acid levels of 97.30 μM (IQR: 68.85–259.2 μM) and 12.65 μM (IQR: 7.65–20.90 μM) were observed in normal diet *Vps33b*^fl/fl^-AlfpCre and *Vps33b*^fl/fl^ samples respectively (Fig.1D, 7.6-fold, *p* = 0.0043, Mann Whitney *U* test). In CA diet *Vps33b*^fl/fl^-AlfpCre and *Vps33b*^fl/fl^ plasma samples, total bile acid levels were increased with respective concentrations of 190.4 μM (IQR: 78.2–295.9 μM) and 24.1 μM (IQR: 13.0–31.9 μM) detected (7.9-fold, *p* = 0.0022).

These results collectively show that key biomarkers of ARC cholestasis (raised plasma ALP and bile acid levels, with normal aminotransferase levels) are recapitulated in *Vps33b*^fl/fl^-AlfpCre mice fed either normal chow or a 0.5% CA diet. As CA feeding did not create artefactual phenotypes in *Vps33b*^fl/fl^-AlfpCre mice and only exacerbated disease phenotypes already observed at physiological level, CA feeding was not considered a confounding factor. The remaining experiments in this study were carried out in CA fed animals unless otherwise stated.

### Histologic findings in livers of *Vps33b*^fl/fl^-AlfpCre mice

Light microscopy of 14-week-old, CA fed *Vps33b*^fl/fl^-AlfpCre mouse liver sections primarily revealed fibrotic changes that were not observed in *Vps33b*^fl/fl^ sections. By trichrome staining (n = 3) we observed periportal and pericentral fibrous expansion with focal bridging fibrosis in *Vps33b*^fl/fl^-AlfpCre mice (Fig.1E). As leucocyte infiltration was observed in *Vps33b*^fl/fl^-AlfpCre liver (Supplementary Fig. 2), these fibrotic changes may indicate a response to inflammation caused by cholestasis in *Vps33b*^fl/fl^-AlfpCre animals.

Trichrome staining of liver sections from 12-month-old *Vps33b*^fl/fl^-AlfpCre mice fed normal chow revealed marked geographic fibrosis and focal steatosis (n = 3) (Fig.1E). This suggests a progression of fibrotic changes and possible defects of lipid metabolism and accumulation in aged animals.

To investigate the mechanism of liver damage in *Vps33b*^fl/fl^-AlfpCre mice, levels of apoptosis were assessed by immunostaining 14-week-old liver sections to detect cleaved caspase 3 (CC3) protein (n = 3). The percentage of CC3 positive cells per field of view was significantly increased in *Vps33b*^fl/fl^-AlfpCre sections in comparison to *Vps33b*^fl/fl^ sections under both normal diet and CA diet conditions (*p* <0.0001 and *p* = 0.0058 respectively, Mann Whitney *U* test) (Supplementary Fig. 3). This suggests that apoptosis may contribute to the liver injury observed in *Vps33b*^fl/fl^-AlfpCre mice. However, in comparison to cholestatic mouse models of bile salt export pump (BSEP) deficiency, the percentage of apoptotic cells detected in *Vps33b*^fl/fl^-AlfpCre sections was relatively low [24].

### *Vps33b*^fl/fl^-AlfpCre animals show mislocalisation of apical membrane proteins

**Fig. 2:**
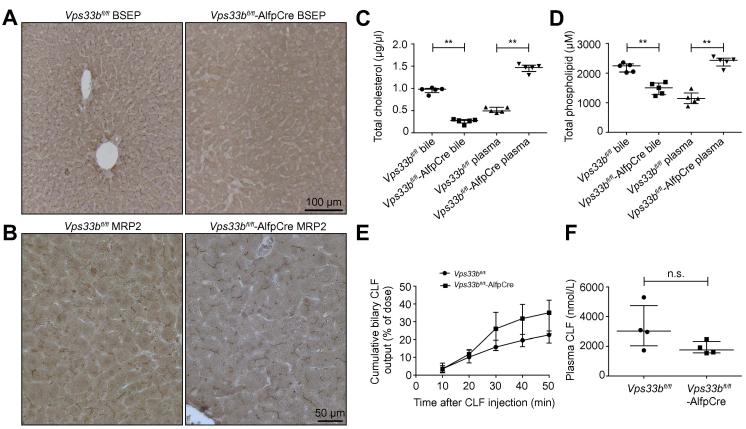
**Functional polarity defects in *Vps33b*^fl/fl^-AlfpCre mice.** (A) Immunostaining for detection of BSEP and (B) MRP2 localisation in *Vps33b*^fl/fl^-AlfpCre and *Vps33b*^fl/fl^ liver sections. (C) Plasma and bile levels of cholesterol and (D) phospholipid in *Vps33b*^fl/fl^-AlfpCre and *Vps33b*^fl/fl^ mice assessed by fluorometric analysis (n = 5, *p* = 0.0079 in all tests, Mann Whitney *U* test). (E) Cumulative biliary levels of CLF and (F) blood levels of CLF measured in *Vps33b*^fl/fl^-AlfpCre and *Vps33b*^fl/fl^ (n = 4). All mice were fed a cholic acid supplemented diet and sacrificed at 14–15 weeks of age. Graphs are presented with medians and IQR.

*In vitro* studies have suggested that VPS33B has a role in apical membrane protein trafficking in epithelial cells [11]. This is reported specifically to involve interaction with Rab11a-positive ARE. To investigate possible defects of protein trafficking to the canalicular membrane, we used immunostaining of *Vps33b*^fl/fl^ and *Vps33b*^fl/fl^-AlfpCre mouse liver sections to assess the localisation of apical membrane proteins known to be trafficked via Rab11a-positive ARE (n = 3). In contrast to *Vps33b*^fl/fl^ tissue, in *Vps33b*^fl/fl^-AlfpCre sections we observed absence of the ATP-binding cassette proteins BSEP and ABCG8 at the hepatocyte canalicular membrane (Fig.2A and Supplementary Fig. 4A). These proteins are responsible for secretion into bile of conjugated bile acids and cholesterol respectively [25], [26], [27]. We also observed mislocalisation of the GPI anchored glycoprotein carcinoembryonic antigen (CEA) in *Vps33b*^fl/fl^-AlfpCre liver with a diffuse stain appearing around the entire hepatocyte membrane as opposed to the clear and selective apical marking in *Vps33b*^fl/fl^ liver (Supplementary Fig. 4B). Multidrug resistance-associated protein 2 (MRP2) reportedly bypasses the RE during apical trafficking [28], [29]. Interestingly, as in ARC patients, MRP2 did not appear mislocalised in *Vps33b*^fl/fl^-AlfpCre mouse liver (Fig.2B). Localisation of basolateral proteins appeared unaffected by loss of *Vps33b* (Supplementary Fig. 4C).

Immunofluorescence detection of BSEP in normal chow fed mice established that protein mislocalisation was not an artefact of CA feeding and also showed intracellular punctate staining in *Vps33b*^fl/fl^-AlfpCre sections (Supplementary Fig. 5A). Immunoblotting analysis showed that BSEP and ABCG8 expression levels were not strongly affected in *Vps33b*^fl/fl^-AlfpCre liver (Supplementary Fig. 5B and C). These data collectively suggest that canalicular proteins were mislocalised to intracellular vesicles rather than downregulated in *Vps33b*^fl/fl^-AlfpCre liver.

### Disruption of functional hepatocyte polarity in *Vps33b*^fl/fl^-AlfpCre mice

To investigate the physiological impact of apical protein mislocalisation, levels of bile components were assessed by mass spectrometry in plasma and bile samples of CA fed *Vps33b*^fl/fl^ and *Vps33b*^fl/fl^-AlfpCre mice (n = 4–6). We observed a significant increase in plasma bile salt levels of *Vps33b*^fl/fl^-AlfpCre animals over those of *Vps33b*^fl/fl^ mice (Table 1). The major accumulating bile acid in plasma was taurocholate, which in *Vps33b*^fl/fl^-AlfpCre mice was present at a median concentration of 97.9 μM: 49-fold higher than the median concentration in *Vps33b*^fl/fl^ mice. The median concentrations of most other bile acid species were also increased: glycocholate 9.7-fold; cholate 7-fold; tauro-diOH-cholanoates 7.5-fold; glyco-diOH-cholanoates 9.3-fold; tauro-tetraOH-cholanoates 80-fold; glyco-tetraOH-cholanoates 26-fold.

In comparison to *Vps33b*^fl/fl^ mice, the main biliary bile acid, taurocholate, was present at 1.9-fold higher concentrations in *Vps33b*^fl/fl^-AlfpCre mouse bile (not statistically significant). Biliary levels of glyco-diOH-cholanoates were equivalent between *Vps33b*^fl/fl^-AlfpCre and *Vps33b*^fl/fl^ animals (Table 2). However, median biliary concentrations of all other bile acids were lower in *Vps33b*^fl/fl^-AlfpCre mice than in *Vps33b*^fl/fl^ mice (1.9-, 13.3-, 1.5- and 4.3-fold reduction in glycocholate, unconjugated cholate, tauro-diOH-cholanoates, and unconjugated diOH-cholanoates respectively). These reduced concentrations were not statistically significant excepting unconjugated cholate (*p* = 0.0190).

Total cholesterol and phospholipid levels were also significantly increased in plasma (2.8- and 2-fold respectively) and decreased in bile (3.6- and 1.5-fold respectively) of *Vps33b*^fl/fl^-AlfpCre animals (*p* = 0.0079 in all tests, Mann Whitney *U* test) (Fig.2C and D). Collectively these results suggest that the directional flow of bile components from blood to canalicular bile is disrupted in *Vps33b*^fl/fl^-AlfpCre mice. Increased levels of plasma total cholesterol were confirmed in normal chow fed *Vps33b*^fl/fl^-AlfpCre (Supplementary Fig. 6A).

In a further group of 14-week-old CA fed animals, we analysed the composition of plasma cholesterol as concentrations of total (Supplementary Fig. 6B), free and esterified cholesterol (n = 6–7). Levels of free cholesterol in plasma of *Vps33b*^fl/fl^-AlfpCre mice were significantly increased (3.6-fold; *p* = 0.0012, Mann Whitney *U* test) over those of *Vps33b*^fl/fl^ animals (Supplementary Fig. 6C, median: 0.3678 μg/μl; IQR: 0.2996–0.5072 μg/μl and median: 0.1036 μg/μl; IQR: 0.0836–0.1615 μg/μl respectively) whilst levels of esterified cholesterol were equivalent between *Vps33b*^fl/fl^-AlfpCre and *Vps33b*^fl/fl^ mice (Supplementary Fig. 6D, median: 0.5134 μg/μl; IQR: 0.3869–0.5459 μg/μl and median: 0.4371 μg/μl; IQR: 0.3998–0.5747 μg/μl respectively). This suggests that the increased levels of total cholesterol observed in plasma of *Vps33b*^fl/fl^-AlfpCre mice are largely due to the increase in concentration of free cholesterol as opposed to esterified cholesterol.

CLF is secreted into canalicular bile specifically via MRP2 [18]. To determine whether MRP2 protein was functional as well as correctly localised in *Vps33b*^fl/fl^-AlfpCre hepatocytes, anaesthetised animals were given an intravenous injection of CLF followed by collection of bile and plasma fractions for fluorometric quantification of CLF (n = 4). Plasma and bile levels of CLF did not significantly differ between *Vps33b*^fl/fl^-AlfpCre and *Vps33b*^fl/fl^ mice, suggesting normal MRP2 function in V*ps33b* deficiency (Fig.2E and F). However, accumulation of CLF in bile appeared more rapid in *Vps33b*^fl/fl^-AlfpCre animals. This is consistent with reports that suggest an increase in mouse MRP2 expression levels in liver in response to bile acid retention [30].

### Inflammatory and metabolic pathways are perturbed in *Vps33b*^fl/fl^-AlfpCre liver

To assess differential gene expression in liver following *Vps33b* deletion, we carried out microarray analysis of total RNA isolated from *Vps33b*^fl/fl^ and *Vps33b*^fl/fl^-AlfpCre liver samples (n = 4–6) from mice fed a 0.5% CA diet and sacrificed at 10–14 weeks.

**Fig. 3:**
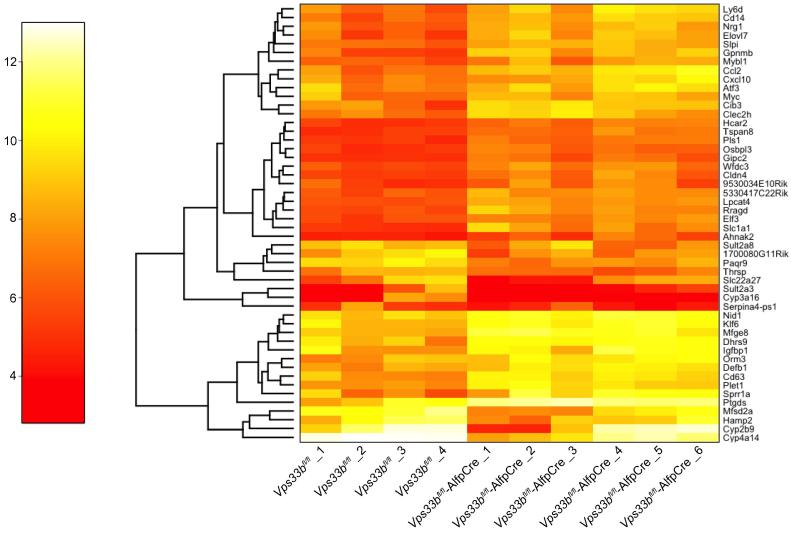
**Gene expression analysis.** Heat map showing gene expression of the top 50 genes identified as differentially expressed by PLS loading in the first component. Analysis was performed on liver RNA samples from 10–14-week-old, cholic acid fed *Vps33b*^fl/fl^ and *Vps33b*^fl/fl^-AlfpCre mice. Color Key: 4 (red) = lower expression level; 12 (yellow) = higher expression level. Left hand trees link genes with similar expression patterns but do not necessarily infer a functional relationship.

Using partial least squares (PLS) regression, we observed that the first PLS component clearly separated *Vps33b*^fl/fl^-AlfpCre from *Vps33b*^fl/fl^ liver samples (Supplementary Fig. 7). Relevant genes were identified by selecting those with the largest contribution to the first component (Fig. 3).

In comparison to samples from Cre-negative littermates, in *Vps33b*^fl/fl^-AlfpCre liver, we observed upregulation of several genes involved in the inflammatory response. This included markers of immune cells (*Ly6d*, *Cd14*) as well as chemokines that induce immune cell infiltration (*Ccl2, Cxcl10*). Using the Database for Annotation, Visualization and Integrated Discovery (DAVID), we identified many over-represented gene ontology annotations in the top 150 genes (Supplementary Fig. 8). In particular, we observed terms relating to ‘inflammatory response’ but annotations such as ‘membrane’ and ‘extracellular region’ were also elicited.

Additionally, numerous genes involved in metabolic pathways were identified as misregulated in *Vps33b*^fl/fl^-AlfpCre mouse liver. Gene set enrichment analysis consistently identified the Kegg pathway bile acid biosynthesis as the most perturbed pathway in *Vps33b*^fl/fl^-AlfpCre animals. Several other metabolic and inflammatory pathways also were perturbed (Supplementary Table 1).

Collectively, these data support our observations of inflammation and fibrotic changes in *Vps33b*^fl/fl^-AlfpCre liver and suggests that intrahepatic retention of bile components may directly result in dysregulation of bile acid metabolism in *Vps33b*^fl/fl^-AlfpCre mice.

### The hepatocyte phenotype is rescued by AAV mediated gene transfer

**Fig. 4:**
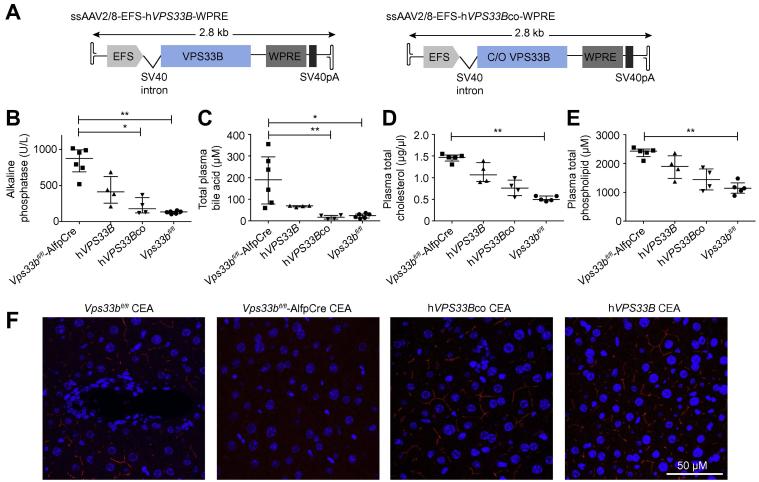
***In vivo* gene transfer of human *VPS33B*.** (A) Schematic of ssAAV2/8 vectors containing wild-type (*hVPS33B*) and codon optimised (*hVPS33Bco*) human *VPS33B* cDNA. (B) Plasma alkaline phosphatase activity levels (*p* = 0.0016, Kruskal-Wallis test), (C) total plasma bile acid levels (*p* = 0.0015, Kruskal-Wallis test) (D) Plasma cholesterol levels (*p* = 0.0023, Kruskal-Wallis test) (E) plasma phospholipid levels (*p* = 0.0047, Kruskal-Wallis test) and (F) CEA protein localisation assessed 9 weeks after injection of ssAAV2/8-EFS-*hVPS33B*-WPRE and ssAAV2/8-EFS-*hVPS33Bco*-WPRE (1 × 10^12^ vg/mouse) in 5-week-old *Vps33b*^fl/fl^-AlfpCre mice (n = 4–6). All mice were fed cholic acid supplemented diet and sacrificed at 14 weeks of age. Graphs are presented with medians and IQR.

To determine whether reintroduction of VPS33B to *Vps33b*^fl/fl^-AlfpCre hepatocytes could restore structural and functional polarity, we performed gene transfer experiments using ssAAV2/8 vectors containing either wild-type or codon optimised human *VPS33B* cDNA (ssAAV2/8-EFS-*hVPS33B*-WPRE and ssAAV2/8-EFS-*hVPS33Bco*-WPRE respectively) (Fig.4A). Vectors were administered to 5-week-old mice at a dose of 1 × 10^12^ vector genomes (vg) per animal via intraperitoneal injection. At 8 weeks of age, injected animals and control groups received CA supplemented feed for 6 weeks. At 14 weeks, without re-exposure to a normal diet, animals were sacrificed and transgene expression in hepatocytes was confirmed immunohistochemically (Supplementary Fig. 9).

In animals treated with ssAAV2/8-EFS-*hVPS33B*-WPRE and ssAAV2/8-EFS-*hVPS33Bco*-WPRE vectors, we observed respective 2.1- and 4.9-fold decreases in median levels of plasma ALP *vs. Vps33b*^fl/fl^-AlfpCre mice (Fig.4B). This was accompanied by a 2.7-fold decrease of total plasma bile acid levels in ssAAV2/8-EFS-*hVPS33B*-WPRE treated mice and a significant 11-fold decrease in plasma bile acid levels of mice receiving ssAAV2/8-EFS-*hVPS33Bco*-WPRE (*p* = 0.0015, Kruskal-Wallis test) (Fig.4C). Plasma levels of cholesterol and phospholipid were also reduced in mice receiving ssAAV2/8-EFS-*hVPS33B*-WPRE and ssAAV2/8-EFS-*hVPS33Bco*-WPRE vectors (Fig.4D and E).

Immunostaining demonstrated partial restoration of canalicular membrane protein localisation following gene transfer with both vectors (Fig.4F; Supplementary Fig. 10). This suggests that, following gene transfer, functional polarity is rescued, as manifest by correction of protein localisation to the apical membrane.

### *Vps33b*^fl/fl^-AlfpCre hepatocytes show tight junction defects

**Fig. 5:**
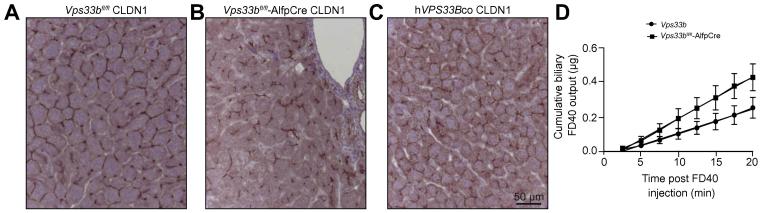
**Tight junction defects in *Vps33b*^fl/fl^-AlfpCre mice.** Appearance of hepatocellular tight junctions assessed by immunostaining for Claudin1 in (A) *Vps33b*^fl/fl^ and (B) *Vps33b*^fl/fl^-AlfpCre mouse liver sections and in (C) sections of *Vps33b*^fl/fl^-AlfpCre mouse liver 9 weeks after injection of ssAAV2/8-EFS-*hVPS33Bco*-WPRE vector (1 × 10^12^ vg/mouse injected at 5 weeks of age) (n = 3). (D) Cumulative FD-40 levels measured in bile fractions from *Vps33b*^fl/fl^-AlfpCre and *Vps33b*^fl/fl^ mice collected after intravenous FD-40 injection (n = 4–5, *p* = 0.0134). All mice were fed a cholic acid supplemented diet and sacrificed at 14–15 weeks of age.

Hepatic tight junctions constitute the barrier that separates sinusoidal blood from canalicular bile. Following knockdown of Rab11a, WIF-B9 cells form tight junctions that appear morphologically abnormal [31]. To study the role of *Vps33b* in Rab11a dependent canalicular membrane biogenesis, we investigated tight junction abnormalities in *Vps33b*^fl/fl^-AlfpCre liver by immunostaining for Claudin1. In control *Vps33b*^fl/fl^ liver, Claudin1 staining revealed normal belt-like tight junctions (Fig.5A). In *Vps33b*^fl/fl^-AlfpCre tissue, Claudin1 marking was irregular and tortuous, suggesting changes in tight junction organisation (Fig.5B). Following gene transfer of 1 x 10^12^ vg/animal ssAAV2/8-EFS-*hVPS33Bco*-WPRE vector, we observed partial correction of tight junction appearance (Fig.5C).

To assess functional integrity of hepatic tight junctions in *Vps33b*^fl/fl^-AlfpCre and *Vps33b*^fl/fl^ liver, we measured levels of FD-40 in bile following intravenous injection. Disruption of tight junction barrier function results in rapid biliary accumulation of FD-40. Fluorometry documented build-up of FD-40 in the bile of *Vps33b*^fl/fl^-AlfpCre mice earlier than in control *Vps33b*^fl/fl^ littermates (*p* = 0.01342) (Fig.5D). This observation suggests that tight junctions in *Vps33b*^fl/fl^-AlfpCre mice, abnormal in Claudin1 distribution on immunostaining, were defective in function.

### Structural abnormalities of *Vps33b*^fl/fl^-AlfpCre canaliculi

**Fig. 6:**
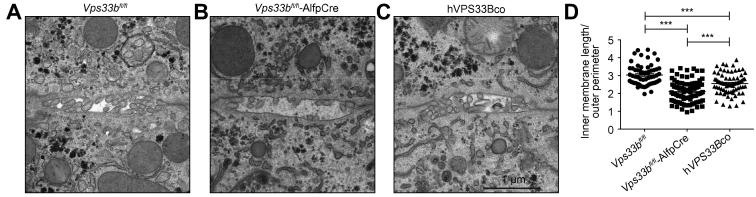
**Bile canaliculus ultrastructure.** Transmission electron micrographs of bile canaliculi in (A) *Vps33b*^fl/fl^ and (B) *Vps33b*^fl/fl^-AlfpCre mouse liver and in (C) liver of *Vps33b*^fl/fl^-AlfpCre mice injected with 1 × 10^12^ vg/mouse of ssAAV2/8-EFS-*hVPS33Bco*-WPRE vector. (D) Measurements of canalicular membrane length (± SEM) as a factor of canalicular perimeter assessed using ITEM software on mouse hepatocyte electron micrographs (n = 62–90, *p* <0.0001, One way ANOVA, Bonferroni’s test). All mice were fed a normal chow diet and sacrificed at 14–15 weeks of age.

Ultrastructure of *Vps33b*^fl/fl^-AlfpCre and *Vps33b*^fl/fl^ canaliculi was assessed by TEM. Relative to Cre-negative littermates, we observed a marked paucity of canalicular microvilli in *Vps33b*^fl/fl^-AlfpCre hepatocytes (Fig.6A and B). Ultrastructure of the bile canaliculi appeared restored in mice treated with ssAAV2/8-EFS-*hVPS33Bco*-WPRE vector (Fig.6C). To quantify these observations in terms of apical membrane length relative to canaliculus size, we measured the total inner membrane length of the bile canaliculi and normalised these values to the length of the outer canalicular perimeter (Fig.6D). In *Vps33b*^fl/fl^-AlfpCre cells we observed a significant decrease in inner membrane length in comparison to Vps33b^fl/fl^ cells. This corresponds to an overall loss of canalicular membrane surface area in the absence of *Vps33b*. This observation was accompanied by a significant increase in canalicular membrane length in hepatocytes of AAV treated mice in comparison to untreated *Vps33b*^fl/fl^-AlfpCre animals (*p* <0.001, One way ANOVA, Bonferroni’s test). Measurements of tight junction length and breadth did not differ between *Vps33b*^fl/fl^ and *Vps33b*^fl/fl^-AlfpCre micrographs (Supplementary Fig. 11).

## Discussion

Loss of apical-basolateral hepatocyte polarity is observed in many types of inherited cholestatic liver disease and in liver cancer [4], [5], [6], [7], [32], [33]. However, the molecular mechanisms that cause this loss are not well determined. In this study, we aimed to improve understanding of the pathways that govern hepatocyte polarity. Specifically, we investigated the role of VPS33B in biogenesis of the bile canaliculus and subsequent maintenance of apical domain structure and function. To achieve this aim, we generated a novel murine model with a liver epithelial cell-specific deletion of *Vps33b* (*Vps33b*^fl/fl^*-AlfpCre*) that recapitulates hepatocyte polarity defects observed in patients with ARC syndrome.

ARC patients with *VPS33B* mutations show cholestasis with high plasma ALP activity and bile acid levels. Patients’ aminotransferase activity and γGT levels remain normal [8]. In agreement with these data, our *Vps33b*^fl/fl^-AlfpCre mice demonstrated significant increases in plasma ALP and total bile acid levels in comparison to Cre-negative littermates. As seen in other cholestasis models, these phenotypes were exacerbated by CA feeding [34]. No significant difference in plasma ALT activity was observed in *Vps33b*^fl/fl^-AlfpCre *vs. Vps33b*^fl/fl^ mice and γGT levels were not assessed as mice do not express γGT in hepatocytes after birth [35]. Biomarkers in our *Vps33b*^fl/fl^-AlfpCre mouse thus accurately track those used to assess ARC liver disease. Like patients with clinically milder ARC, *Vps33b*^fl/fl^-AlfpCre animals did not manifest increased serum bilirubin levels.

As ALP is normally expressed at the apical hepatocyte membrane, the increase in plasma ALP levels in *Vps33b*^fl/fl^-AlfpCre animals may be due to defects in apical membrane biosynthesis. Further increases in plasma ALP caused by the CA diet may be related to canalicular damage, although increased ALP synthesis or shifts in localisation are also possible. Further evidence of canalicular injury is supplied by immunostaining of ARC patient liver biopsy specimens, which reveals mislocalisation of proteins typically located at the apical membrane. Among these is BSEP, an ATP-binding cassette transporter required for movement of conjugated bile acids from blood to bile [25]. The mislocalisation of BSEP was reproduced in our *Vps33b*^fl/fl^-AlfpCre animals under both normal diet and CA diet conditions. By immunofluorescence, we observed punctate intracellular BSEP staining, consistent with localisation of BSEP in intracellular vesicles.

In patients with progressive familial intrahepatic cholestasis type 2 (PFIC2), the loss of BSEP results in intrahepatic retention of bile salts [36]. In the *Bsep* knockout mouse, severe cholestatic liver disease is observed when mice are fed a diet containing 0.5% CA [24]. In agreement with these observations, by utilising a recently developed mass spectrometry technique, we detected significant increases (7 to 80-fold) in plasma concentrations of TCA, GCA, CA, tauro-diOH-cholanoates, glyco-diOH-cholanoates, tauro-tetra-OH-cholanoates and glyco-tetra-OH-cholanoates in *Vps33b*^fl/fl^-AlfpCre mice fed 0.5% CA. Concentrations of plasma bile acids in *Vps33b*^fl/fl^-AlfpCre animals were only slightly lower than values previously observed in the *Bsep* knockout mouse [24].

Our comparison of biliary bile acid concentrations in *Vps33b*^fl/fl^-AlfpCre and *Vps33b*^fl/fl^ mice also yielded results similar to those obtained on comparison of *Bsep* knockout and wild-type mice [24]. Neither the concentration of the major biliary bile acid component, taurocholate, nor the total biliary bile acid concentration differed significantly between *Vps33b*^fl/fl^-AlfpCre and *Vps33b*^fl/fl^ animals. This may indicate an alternative route of apical bile acid secretion following CA feeding such as via MRP2 [37]. For some of the lesser bile acids, biliary concentrations were lower in *Vps33b*^fl/fl^-AlfpCre mice than in *Vps33b*^fl/fl^ mice. This reduction in biliary concentration was statistically significant only in the case of unconjugated cholate. As in the *Bsep* knockout mouse, this may indicate a higher rate of taurine conjugation and conversion to tetra-OH bile acid species in *Vps33b*^fl/fl^-AlfpCre animals as a mechanism of reducing bile acid toxicity [38]. Indeed, the proportion of unconjugated bile acids in plasma and bile of *Vps33b*^fl/fl^-AlfpCre mice was lower than that of *Vps33b*^fl/fl^ littermates and a significant increase in conjugated tetra-OH species was observed in *Vps33b*^fl/fl^-AlfpCre plasma. Overall, these data suggest that mislocalisation of BSEP in *Vps33b*^fl/fl^-AlfpCre mice has a similar effect on transport of bile acids to the effect of *Bsep* knockout. However, even CA fed *Vps33b*^fl/fl^-AlfpCre animals had normal serum bilirubin concentrations, no obvious reduction in survival and only a small increase in apoptosis (as detected by CC3 staining), thus suggesting liver disease less severe than that in *Bsep* knockout mice [24].

In addition to BSEP mislocalisation, in *Vps33b*^fl/fl^-AlfpCre hepatocytes we observed mislocalisation of ABCG8, an apical ATP-binding cassette transporter involved in the directional movement of cholesterol [27]. Concordant with a loss of apical ABCG8, in comparison to *Vps33b*^fl/fl^ mice, we detected reduced levels of total cholesterol in bile and accumulation of cholesterol in plasma of CA fed *Vps33b*^fl/fl^-AlfpCre animals. Collectively these results suggest that, in the absence of *Vps33b*, apical transporter proteins are mislocalised, causing an overall loss of functional polarity and accumulation of bile components in blood.

Interestingly, the increase in plasma total cholesterol observed in *Vps33b*^fl/fl^-AlfpCre animals was due to an increase in free cholesterol as opposed to esterified cholesterol. In combination with our observation of canalicular proteins mislocalised to possible intracellular vesicles, as in other cholestatic models, this increase in free cholesterol may imply that the majority of phospholipid and cholesterol in *Vps33b*^fl/fl^-AlfpCre plasma is present in the form of lipoprotein X, biliary lipid vesicles that contain mostly free cholesterol and phosphatidylcholine, ascribed in cholestasis to shedding of hepatocyte membranes into bile and thence, with bile, between hepatocytes into plasma [39], [40].

Proteins that appear mislocalised in *Vps33b*^fl/fl^-AlfpCre mouse hepatocytes are thought to be trafficked to the canalicular membrane specifically via Rab11a-positive ARE [41]. In our model we observed mislocalisation of apical proteins that are trafficked directly from the trans golgi network (TGN) to the ARE (BSEP and ABCG8) as well as proteins that are first delivered to the basolateral membrane from TGN and then transcytosed to the canalicular membrane via ARE (CEA) [41]. However, as in ARC patients, MRP2 was not mislocalised in *Vps33b*^fl/fl^-AlfpCre liver. MRP2 is reported to bypass Rab11a-positive recycling ARE en route to the hepatocyte apical domain [28], [29]. Functional integrity of MRP2 in *Vps33b*^fl/fl^-AlfpCre liver was confirmed by intravenous administration and subsequent quantification in bile of cholyl-lysyl-fluorescein (a compound specifically exported into bile via MRP2) [18]. As in zebrafish, these results suggest that mammalian VPS33B is specifically involved in trafficking of proteins to the apical domain via Rab11a-positive ARE [11]. Basolateral membrane proteins were not mislocalised in *Vps33b*^fl/fl^-AlfpCre hepatocytes, suggesting a specific role for VPS33B in biogenesis of the bile canaliculus.

As we observed in mutant mice that MRP2 is functional and normally sited, as is MRP2 in ARC patients [11], we infer that the collections of Dubin-Johnson-like pigment in some ARC patients’ hepatocytes [42] are unlikely to originate from MRP2 absence or dysfunction.

To assess defects in structural components of hepatocyte polarity, we analysed tight junction integrity in *Vps33b*^fl/fl^-AlfpCre and *Vps33b*^fl/fl^ liver. As in cultured cells, immunohistochemical marking for Claudin1, a tight junction component, was irregular in *Vps33b*^fl/fl^-AlfpCre liver, with tortuous distribution [31]. Tight junctions between control *Vps33b*^fl/fl^ hepatocytes appeared uniform and belt-like. This suggestion of a structural tight junction defect was supported by our functional analysis of tight junction integrity which showed rapid accumulation of fluorescence in the bile of *Vps33b*^fl/fl^-AlfpCre mice following intravenous injection of 40 kDa fluorescent dextran (FD-40). The observed leaking of FD-40 from blood into bile suggests a loss of tight junction barrier function in *Vps33b*^fl/fl^-AlfpCre hepatocytes [19]. Whether tight junction proteins are normally trafficked to the apical membrane by VPS33B or loss of tight junction integrity is a secondary effect of incorrect apical protein trafficking remains to be determined. In contrast to reports in *Tjp2* knockout mice, our TEM analysis of tight junction ultrastructure revealed no overall increase in tight junction length or breadth following deletion of *Vps33b* [4], [7]. In future studies, freeze fracture electron microscopy may be used for a more detailed assessment of tight junction composition in *Vps33b*^fl/fl^-AlfpCre liver.

Analysis of ultrastructural images of *Vps33b*^fl/fl^-AlfpCre liver revealed a significant reduction in apical membrane area due to a partial loss of canalicular microvilli. This implies a loss of structural polarity at the apical membrane and an overall reduction of surface area for movement of solutes from the hepatocyte into bile. When *Rab11a* is mutated in mouse intestine, apical brush border microvilli are lost [43]. These results provide an interesting link between the observed interaction of VPS33B in Rab11a protein trafficking pathways and the maintenance of apical microvilli in hepatocytes.

To confirm the role of VPS33B in hepatocyte polarity, we performed *in vivo* gene rescue studies in *Vps33b*^fl/fl^-AlfpCre mice using ssAAV2/8 vectors containing either wild-type or codon optimised human *VPS33B* cDNA (ssAAV2/8-EFS-*hVPS33B*-WPRE and ssAAV2/8-EFS-*hVPS33Bco*-WPRE). At 9 weeks after gene transfer (1 × 10^12^ vg/mouse in 4–5 week old animals) we observed partial rescue of apical protein localisation, Claudin1 distribution at tight junctions, canalicular ultrastructure and plasma ALP activity, cholesterol, phospholipid and bile acid levels. This phenotype correction appeared more robust in animals treated with the codon optimised vector: all such animals had plasma total bile acid levels equivalent to those in control *Vps33b*^fl/fl^ mice. As well as confirming that the observed loss of polarity in *Vps33b*^fl/fl^-AlfpCre hepatocytes is due to *Vps33b* mutation, these experiments establish an initial proof of principle for future therapeutic use of gene transfer in ARC patients. To meet a therapeutic aim, dose optimisation and safety of *VPS33B* gene transfer would need to be determined in future studies.

In conclusion, using our novel *Vps33b*^fl/fl^-AlfpCre mouse model, we have identified that VPS33B is vital for maintenance of mammalian structural and functional hepatocyte polarity. We have established that genetic hepatocyte polarity disorders can be rescued using gene transfer with AAV vectors and that ARC liver disease may be a potential target of therapeutic gene replacement.

## Financial support

Children’s Liver Disease Foundation (CLDF), Great Ormond Street Hospital and UCL Institute of Child Health Biomedical Research Centre. European Research Council (grant ref. ERC-2013-StG-337057 CLOC), P.G. is a Welcome Trust senior clinical research fellow. J.J.B. is supported by core funding to MRC_UCL LMCB university unit (grant ref. MC_U122665002).

## Conflict of interest

Dr. Clayton reports grants and personal fees from Actelion Pharmaceuticals UK, outside the submitted work. All the other authors who have taken part in this study declared that they do not have anything to disclose regarding funding or conflict of interest with respect to this manuscript.

## Authors’ contributions

J.H. performed experiments, contributed to overall study design and wrote the manuscript which was edited by all co-authors. D.D., F.M., J.J.B., R.F., H.S., A.S-I., B.B., A.V. and S.W. performed experiments. A.M-L. performed *in silico* gene expression and pathway analysis. D.D., J.J.B., K.M., F.P.L., A.S.K., S.H., N.S., S.W., C.C.P., P.C. and P.G. contributed to overall study design. P.G. directed the study.

## Figures and Tables

**Table 1:** **Concentrations of bile acids in plasma from *Vps33b*^fl/fl^ and *Vps33b*^fl/fl^-AlfpCre mice fed 0.5% cholic acid.**

**Bile acid**	***Vps33b*^fl/fl^**			***Vps33b*^fl/fl^-AlfpCre**			**Comparison**
	**Median,** μ**M**	**25th Percentile**	**75th Percentile**	**Median,** μ**M**	**25th Percentile**	**75th Percentile**	***p* value** **(Mann Whitney *U*)**
TCA	1.99 (9.69%)	1.49	4.16	97.9 (54.55%)	46.2	183	0.002
GCA	1.09 (5.31%)	0.57	1.61	10.6 (5.91%)	6.99	24.4	0.002
CA	3.44 (16.75%)	2.19	5.84	24.2 (13.48%)	5.50	33.0	0.02
Tauro-diOH-C	1.98 (9.64%)	1.28	2.62	14.8 (8.25%)	5.67	25.4	0.004
Glyco-diOH-C	0.08 (0.39%)	0.038	0.15	0.74 (0.41%)	0.20	2.30	0.002
DiOH-C	11.8 (57.45%)	5.41	19.9	24.0 (13.37%)	11.8	33.4	0.13 (ns)
Tauro-tetraOH-C	0.08 (0.39%)	0.068	0.16	6.40 (3.57%)	1.77	10.8	0.0048
Glyco-tetraOH-C	0.03 (0.15%)	0.018	0.057	0.78 (0.43%)	0.64	2.68	0.0238
TetraOH-C	0.05 (0.23%)	0.03	0.058	0.06 (0.03%)	0.03	0.09	0.5097 (n.s.)

Contributions of individual bile acids to the total bile acid pool are listed as percentages in parenthesis.

CA, cholic acid; C, cholanoate; T, tauro; G, glyco; DiOH, dihydroxy; tetra-OH, tetrahydroxy.

**Table 2:** **Concentrations of bile acids in bile from *Vps33b*^fl/fl^ and *Vps33b*^fl/fl^-AlfpCre mice fed 0.5% cholic acid corrected by flow rate.**

**Bile acid**	***Vps33b^fl/fl^***			***Vps33b^fl/fl^-*AlfpCre**			**Comparison**
	**Median (nmol/min.100 g)**	**25th Percentile**	**75th Percentile**	**Median (nmol/min.100 g)**	**25th Percentile**	**75th Percentile**	***p* value** **(Mann Whitney *U*)**
TCA	47.26 (68.48%)	33.76	53.74	90.42 (92.26%)	70.67	99.24	0.0667(n.s.)
GCA	2.93 (4.25%)	2.12	3.99	1.58 (1.61%)	0.98	2.88	0.2571 (n.s.)
CA	11.21 (16.24%)	7.28	11.60	0.84 (0.86%)	0.14	4.55	0.0190
Tauro-diOH-C	7.30 (10.58%)	4.96	8.41	4.93 (5.03%)	2.74	6.23	0.0667(n.s.)
Glyco-diOH-C	0.18 (0.26%)	0.15	0.21	0.21 (0.21%)	0.19	0.27	0.1714 (n.s.)
DiOH-C	0.13 (0.19%)	0.08	0.31	0.03 (0.03%)	0.00	0.08	0.1143(n.s.)

Contributions of individual bile acids to the total bile acid pool are listed as percentages in parenthesis.

CA, cholic acid; C, cholanoate; T, tauro; G, glyco; DiOH, dihydroxy; tetra-OH, tetrahydroxy.
