# Supplementary material for: Vps33b is crucial for structural and functional hepatocyte polarity
Source: J Hepatol. 2017 May;66(5):1001–11. doi: 10.1016/j.jhep.2017.01.001 (PMC5387182; doi:10.1016/j.jhep.2017.01.001)
Supplement: Supplementary data 2 [file mmc1.pdf]

Vps33b is crucial for structural and functional hepatocyte polarity

Joanna Hanley, Dipok Kumar Dhar, Francesca Mazzacuva, Rebeca Fiadeiro, Jemima J. Burden, Anne-Marie Lyne, Holly Smith, Anna Straatman-Iwanowska, Blerida Banushi, Alex Virasami, Kevin Mills, Frédéric P. Lemaigre, AS Knisely, Steven Howe, Neil Sebire, Simon Waddington, Coen C. Paulusma, Peter Clayton, Paul Gissen

Table of Contents

Supplementary methods 2
- Immunoblotting 2
- Mass spectrometry........ 3
- Histology 4
- Transmission electron microscopy 5
- Serum and bile biochemical assays 5
- In silico gene expression and pathway analysis 6
- Molecular cloning for AAV vector production 6

Supplementary Figures 7
- Supplementary Fig.1. Confirmation of Vps33b gene deletion. 7
- Supplementary Fig.2. Leucocyte infiltration in *Vps33b*$^{fl/fl}$*-AlfpCre* liver. 8
- Supplementary Fig.3. Cleaved Caspase 3 quantification. 9
- Supplementary Fig.4. Protein localisation. 10
- Supplementary Fig.5. Confirmation of protein mislocalisation. 11
- Supplementary Fig.6. Analysis of plasma cholesterol composition. 12
- Supplementary Fig.7. PLS scores. 13
- Supplementary Fig.8. GO annotations. 14
- Supplementary Fig.9. Expression of VPS33B following gene transfer. 15
- Supplementary Fig.10. Gene transfer restores apical BSEP localisation. 16
- Supplementary Fig.11. Tight junction ultrastructure. 17

Supplementary Tables 18

Supplementary Table 1. Pathways disrupted by loss of Vps33b expression in liver. 18
Supplementary Table 2. Antibody list. 19
References 19

Supplementary methods

Immunoblotting

Immunoblotting was performed on whole liver protein extracts or total membrane protein fractions. Total membrane proteins were isolated from liver tissue using the MEM-Per Plus Membrane Protein Extraction Kit per manufacturer' instructions (Thermo Fisher Scientific, Loughborough, UK). Protein concentrations, in total liver and membrane extracts, were quantified using the BCA Protein Assay Kit (Thermo Fisher Scientific, Loughborough, UK) per manufacturer' instructions. Samples from total liver were heated in loading buffer at 95°C for 5 min prior to loading 20μg of each sample into wells of 7.5% Tris-Glycine gels (Bio-Rad, Hertfordshire, UK). Membrane extracts (15μg per sample) were loaded directly into wells of 7.5% Tris-Glycine gels in loading buffer without heating. Samples were run at 120V constant for 50 min in TRIS/GLYCINE/SDS running buffer (Geneflow, Staffordshire, UK). Proteins were transferred to polyvinylidene difluoride membranes at 0.25A constant for 1hr in 20% Methanol TRIS/GLYCINE transfer buffer (Geneflow, Staffordshire, UK). Membranes were then blocked in blocking buffer (PBST-5% milk) for 30 min at room temperature prior to incubation in primary antibody (diluted in blocking buffer) overnight at 4°C. Membranes were washed in PBST and incubated in secondary antibody (diluted in blocking buffer) for 1hr at room temperature. Membranes were washed 3 times with PBST followed by incubation with enhanced chemiluminescence substrate (ECL Prime, GE Healthcare, Buckinghamshire, UK).

Protein bands were visualised using a charge-coupled device imager (ImageQuant LAS 4000mini, GE Healthcare, Buckinghamshire, UK).

Mass spectrometry

Cholic acid (CA), chenodeoxycholic acid (CDCA) and deoxycholic acid (DCA) and their glycine and taurine conjugates (GCA, TCA, GCDCA, TCDCA, GDCA, TDCA) were purchased from Steraloids (Newport, RI) while all deuterium labelled internal standards used (D_4-CA, D_4-CDCA, D_4-GCA, D_5-TCA, D_4-GCDCA and D_4-TCDCA) were from CDN Isotopes (Pointe-Claire, Quebec, Canada). For bile samples, 10µL of bile was spotted onto neonatal screening cards (Whatman 903TM, GE Healthcare, Amersham, UK). The whole sample was punched out and eluted by sonication with 300µL of methanol containing the deuterated bile acid internal standards (20nM) (1). Samples were further diluted 1:100 (or more when required) before UPLC-MS/MS analysis. For plasma samples, protein precipitation/bile acid extraction was conducted by adding 140µL of methanol containing the deuterated bile acid internal standards (final concentration 20nM) to 10µL of plasma. Supernatant was recovered after centrifugation (5 min; 13000 rpm). Because such chromatography does not fully separate DCA from CDCA, GDCA from GCDCA and TDCA from TCDCA, in each case, the combined peak was integrated, with results reported as dihydroxycholanoates (diOH-C), glycodihydroxycholanoates (glyco-diOH-C) and tauro-diOH-C or tetrahydroxycholanoates (tetraOH-C), glycotetrahydroxycholanoates (glycol-tetraOH-C) and taurotetrahydroxycholanoates (tauro-tetraOH-C) respectively.

Histology

Livers were fixed in 4% paraformaldehyde or 10% neutral-buffered formalin and embedded in paraffin. Sections were trichrome or haematoxylin and eosin (H&E) stained by the Great Ormond Street Hospital, Camelia Botnar Laboratories histology services or processed for immunohistochemistry/immunofluorescence (IHC/IF). For immunostaining, sections were deparaffinised and incubated in Tris-EDTA buffer at 95°C for 40 min for heat induced epitope retrieval. Sections were blocked in 3% hydrogen peroxide for 15 min at room temperature, blocked in 5% goat serum for 1 hr at room temperature and then incubated overnight at 4°C with primary antibodies. For IF, sections were then incubated for 1 hr at room temperature with Alexa 488 or 594 labelled secondary antibodies; counterstained with 4',6-diamidino-2-phenylindole dihydrochloride and mounted in ProLong Gold Antifade Reagent (Life Technologies, Paisley, UK). For IHC, sections were incubated with biotinylated secondary antibodies for 1 hr at room temperature followed by incubation with VECTASTAIN Elite ABC Kit reagents (Vector Laboratories, Peterborough, UK) per manufacturer' instructions. IHC sections were developed with 3,3′-diaminobenzidine substrate, counterstained with haematoxylin, dehydrated and permanently mounted in DPX mountant. Experienced histopathologists (A.K., N.S.) assessed liver histology in a blinded analysis.

To quantify cleaved Caspase 3 expression, positively stained nuclei were counted. The value obtained was formulated as a percentage of CC3 positive cells per field of view. Nuclei were counted in 10 fields of view per animal (n=3).

Transmission electron microscopy

Immediately after sampling, a portion of liver (approximately 8mm × 8mm × 3mm) was fixed by infiltration injection of warm fixative (1.5% glutaraldehyde, 1% sucrose in 0.1M sodium cacodylate). Once the tissue greyed and firmed (indicating good fixation), it was dissected into smaller pieces (1mm × 1mm × 1mm) and left in fixative for a total of 20 minutes. The samples were then washed several times with 0.1M sodium cacodylate supplemented with 1.5% sucrose, incubated with 1% osmium tetroxide/1.5% potassium ferricyanide for 2 hours at 4˚C, then washed with 0.1M sodium cacodylate and stored at 4˚C until all samples were ready for further processing. Additional pieces of fixed tissue were sliced into 100μm thick slices using a vibrating microtome and processed as above. All samples were treated with 1% tannic acid before ethanol dehydration and embedding in Epon (TAAB, Reading, UK). Ultrathin sections (70 nm) were taken using a Leica UC7 ultramicrotome (Leica Microsystems, Vienna, Austria) and collected on Formvar coated slot grids. Sections were lead citrate stained, then imaged using a Tecnai T12 Spirit Biotwin (FEI, Eindhoven, Netherlands) and Morada CCD camera using iTEM (EMSIS, Münster, Germany). Photomicrographs were assessed by an experienced electron microscopist (J.J.B).

Serum and bile biochemical assays

Total cholesterol, free cholesterol, esterified cholesterol and total phospholipid levels were measured in plasma and bile using commercially available kits per manufacturer' instructions (Sigma Aldrich, Gillingham, UK). Serum alanine aminotransferase, bilirubin and alkaline phosphatase levels were determined using automated methods by the Great Ormond Hospital, Camelia Botnar Laboratories chemical pathology services.

In silico gene expression and pathway analysis

Partial least squares (PLS) regression (2), implemented in the R package pls (3), was used to identify genes with differential expression in knockout mouse livers compared to controls. Genes were ranked based on their loading in the first PLS component, as this component clearly separated knockouts from controls. The online software DAVID (4) was used to look for statistically significant and over-represented Gene Ontology (GO) annotations in the top 150 genes. Gene set enrichment analysis (GSEA), coded in the R statistical environment, was used to identify gene sets with a larger than expected number of differentially expressed genes. The square of the PLS loading in the first component was used to score genes, and the score for each gene set was computed using the mean of all the genes in that set. P-values were computed using permutation.

Molecular cloning for AAV vector production

An expression cassette containing the elongation factor 1α short (EFS) promoter and a modified SV40 small t antigen intron upstream of a codon optimised human *VPS33B* cDNA (*hVPS33Bco*) was synthesised by GenScript (Piscataway, NJ, USA). The cassette was ligated into an ssAAV2 backbone plasmid upstream of the simian virus 40 late polyA (SV40 LpA) as a HindIII-NotI fragment. A WPRE sequence was generated by PCR and ligated downstream of *hVPS33Bco* as an EcoRV-SalI fragment. To obtain an ssAAV2 plasmid containing wild type human *VPS33B* cDNA (*hVPS33B*), h*VPS33B* cDNA generated by PCR was ligated into the pAV-EFS-*hVPS33Bco*-WPRE-SV40 LpA plasmid as a NheI-BamHI fragment.

Supplementary Figures

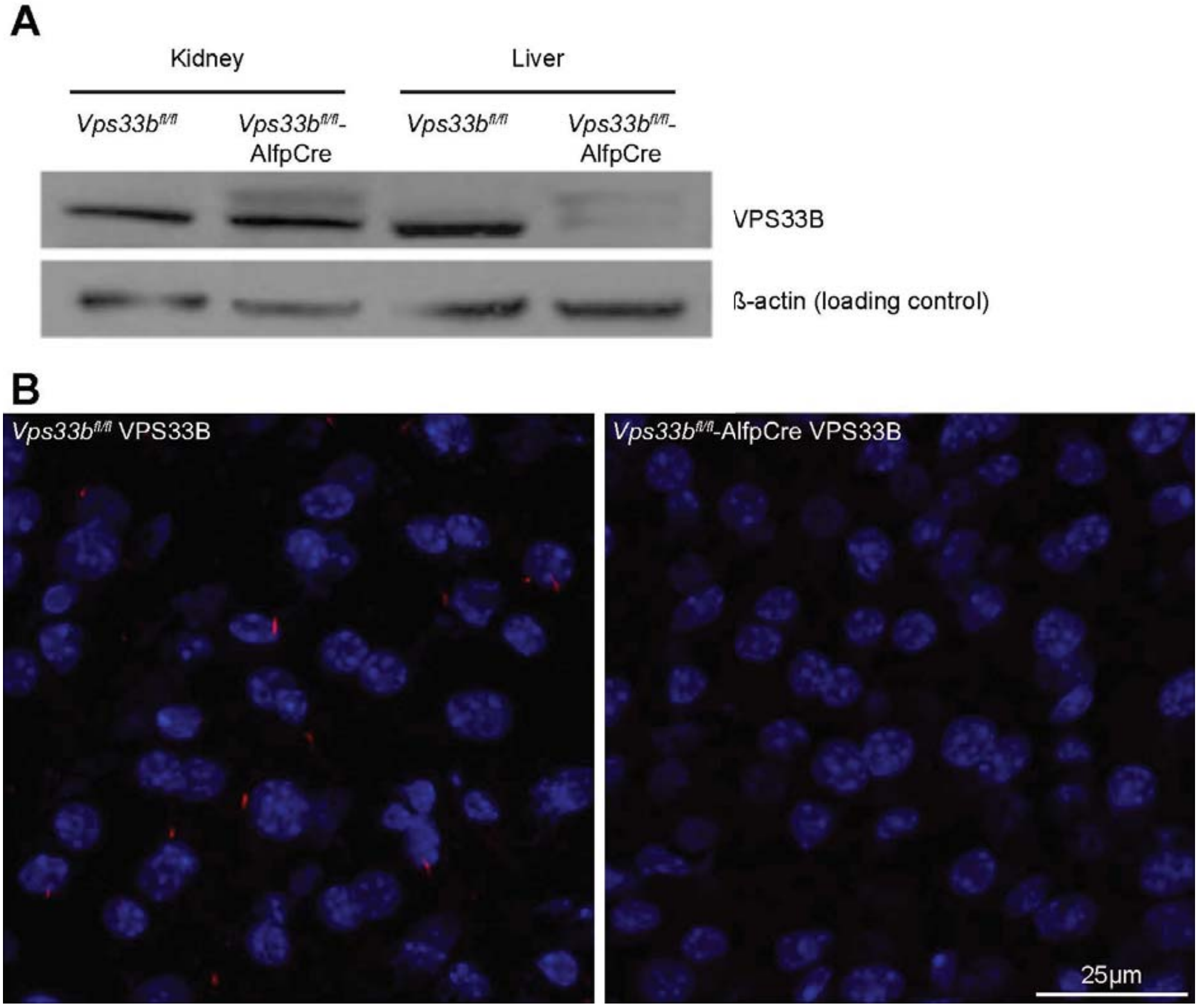

Supplementary Fig.1. Confirmation of Vps33b gene deletion. Liver-specific deletion of *Vps33b* in *Vps33b*$^{fl/fl}$*-AlfpCre* mice assessed by (A) western blotting for VPS33B protein in mouse liver and kidney lysates and (B) fluorescence in situ hybridisation on liver sections from *Vps33b*$^{fl/fl}$*- AlfpCre* and *Vps33b*$^{fl/fl}$ mice (n=3).

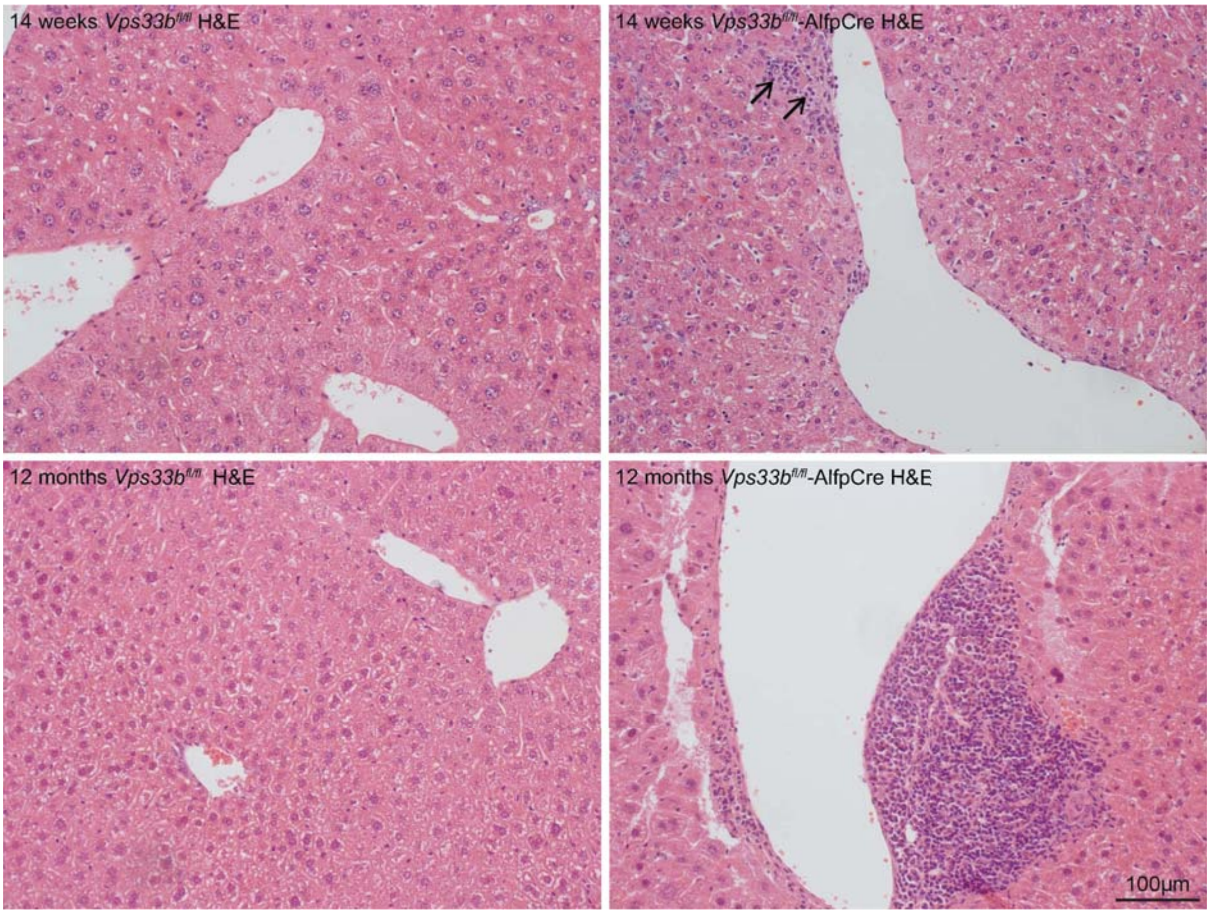

Supplementary Fig.2. Leucocyte infiltration in *Vps33b$^{fl/fl}$-AlfpCre* liver. H&E staining on liver sections from *Vps33b$^{fl/fl}$* and *Vps33b$^{fl/fl}$-AlfpCre* mice aged 14-weeks and fed a cholic acid diet or aged 12 months and fed a normal diet (n=3). Arrows indicate milder regions leucocyte infiltration in 14-week-old *Vps33b$^{fl/fl}$-AlfpCre* liver.

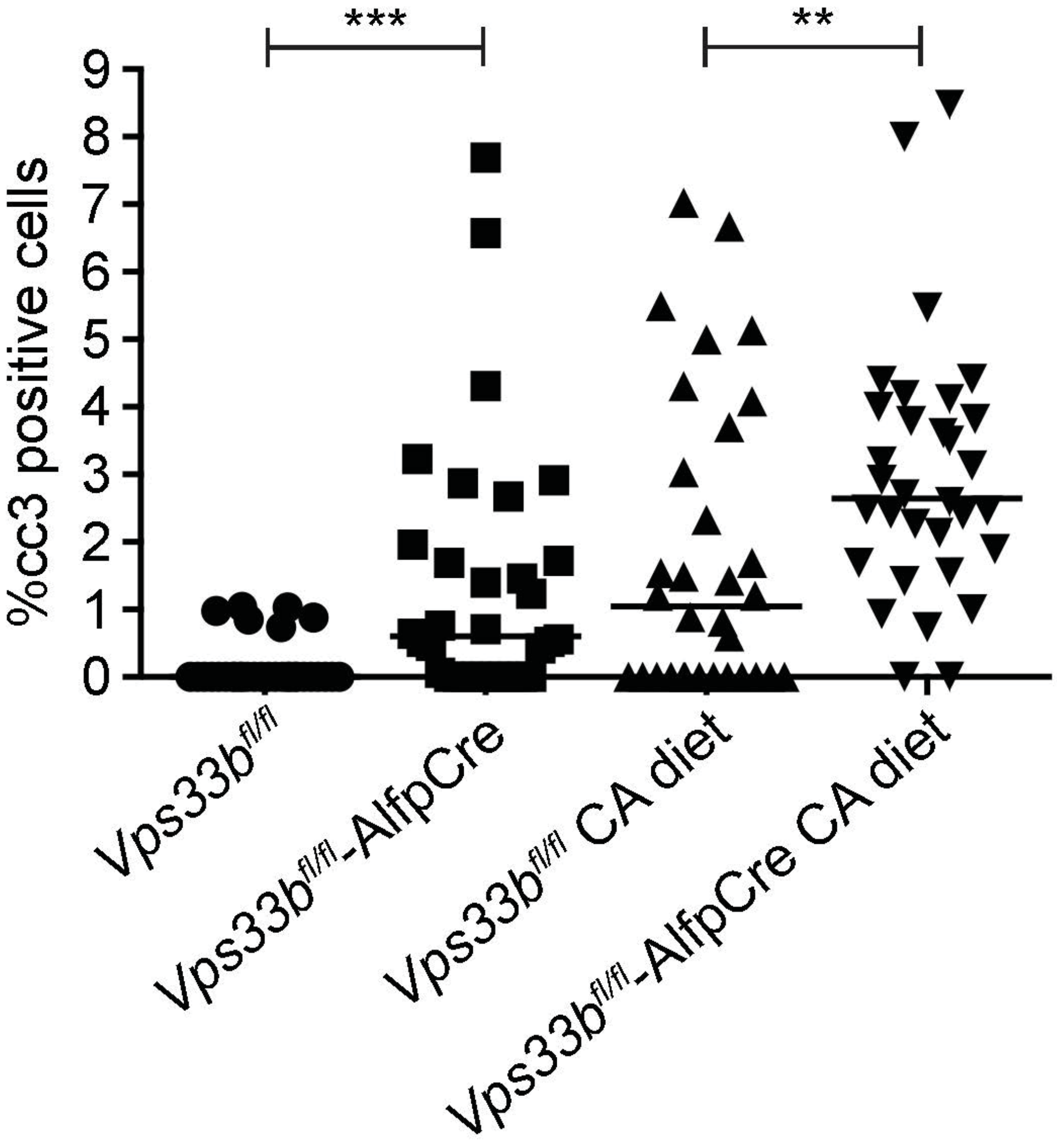

Supplementary Fig.3. Cleaved Caspase 3 quantification. Liver sections from normal chow and CA fed 14-week-old Vps33b$^{fl/fl}$-AlfpCre and Vps33b$^{fl/fl}$ mice were stained for CC3 by immunohistochemistry. Positively stained nuclei were counted and expressed as a percentage of positive cells per field of view. Ten fields of view were counted per animal (n=3). P-values: normal chow, P=<0.0001; CA diet, P=0.0058 (Mann Whitney U test).

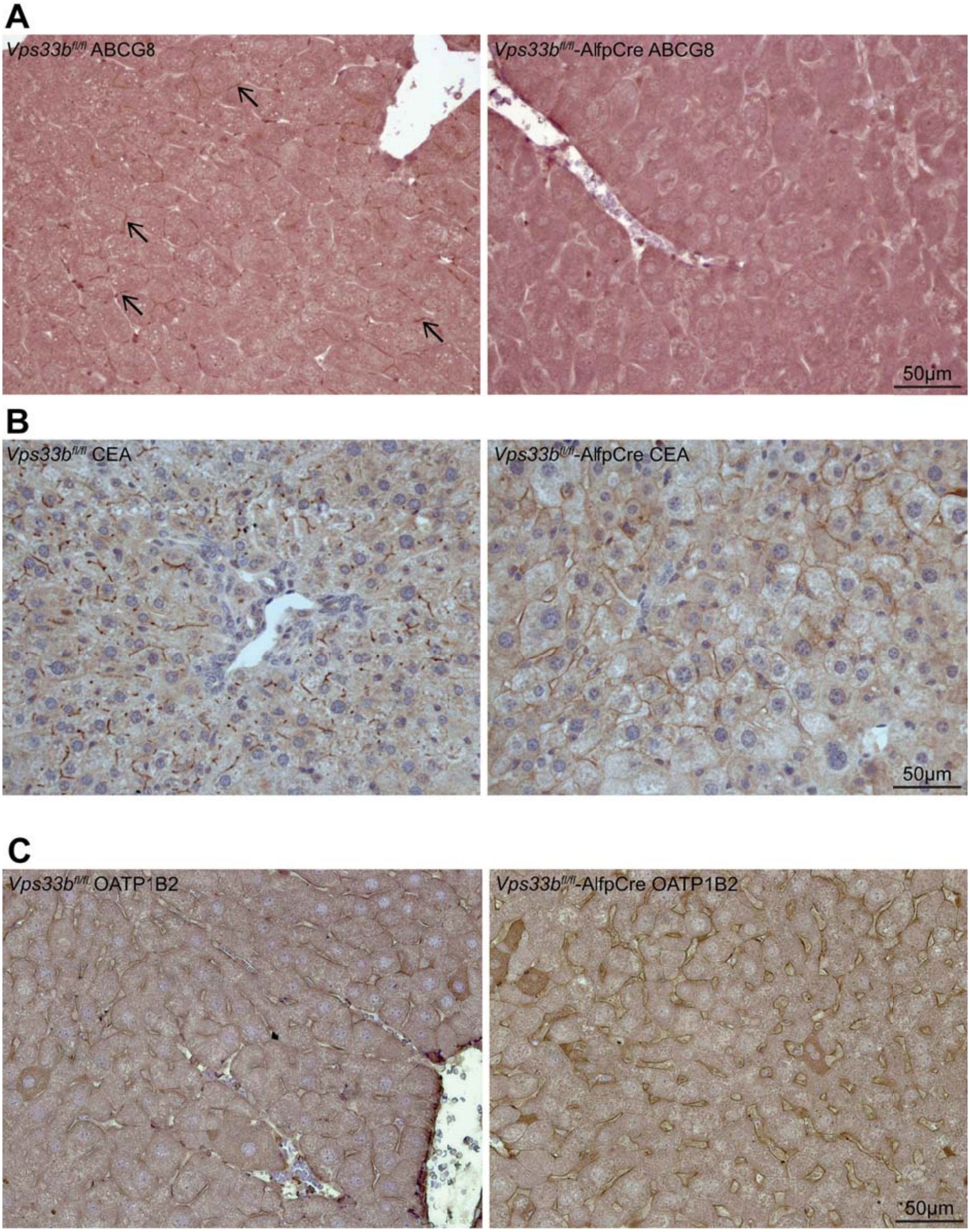

Supplementary Fig.4. Protein localisation. Localisation of (A) ABCG8, (B) CEA and (C) OATP1B2 protein assessed by immunohistochemistry on liver sections from 14-week-old, cholic acid fed *Vps33b*$^{fl/fl}$- *AlfpCre* and *Vps33b*$^{fl/fl}$ mice. Arrows indicate typical regions of apical membrane staining.

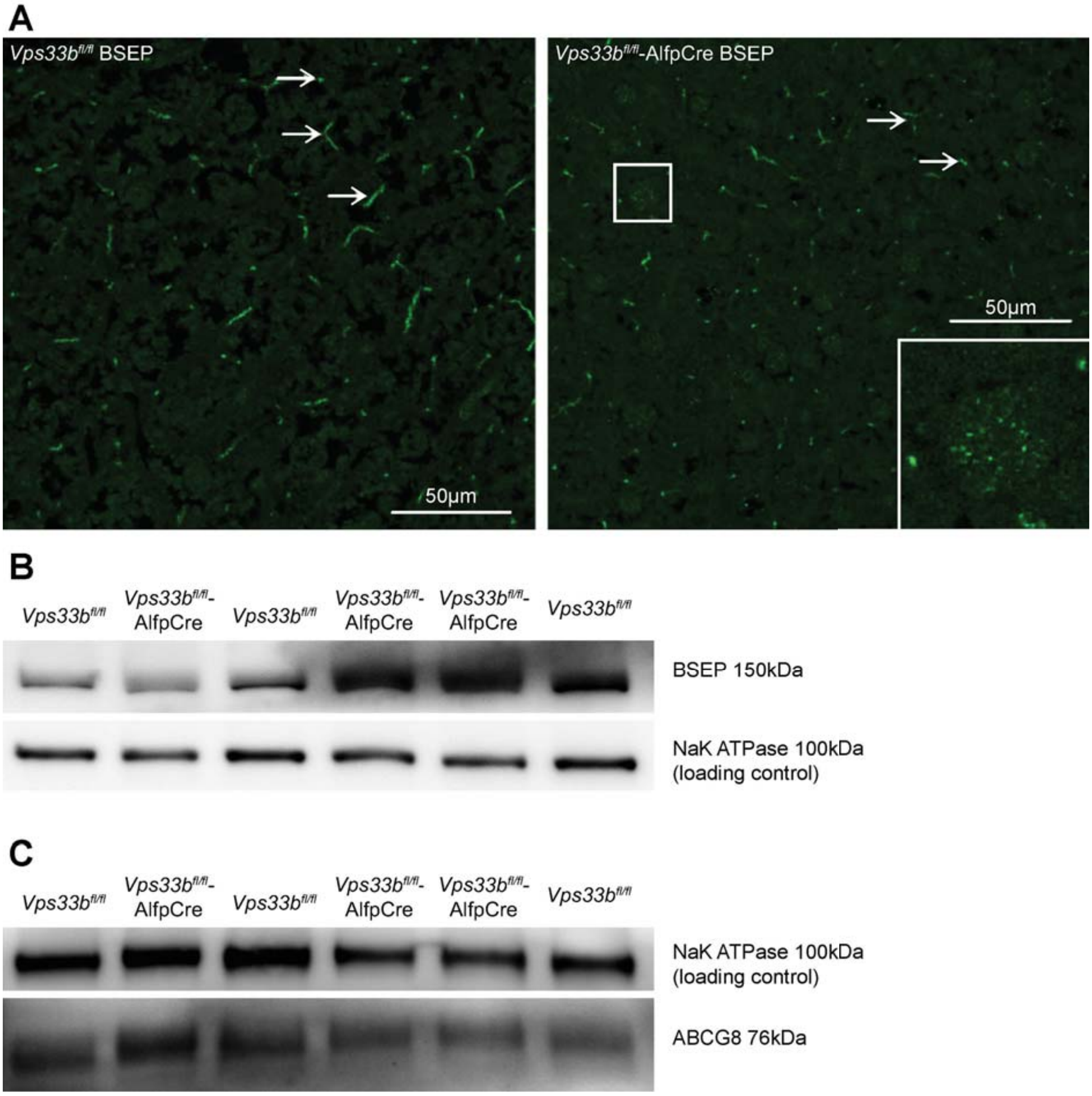

Supplementary Fig.5. Confirmation of protein mislocalisation. (A) Immunofluorescence detection of BSEP protein in Vps33b$^{fl/fl}$-AlfpCre and Vps33b$^{fl/fl}$ liver sections confirmed apical protein mislocalisation in 14-week-old Vps33b$^{fl/fl}$-AlfpCre mice fed normal chow. The zoomed in image shows possible mislocalisation of BSEP to intracellular vesicles in Vps33b$^{fl/fl}$-AlfpCre liver. Arrows indicate regions of apical staining that appear more punctate in Vps33b$^{fl/fl}$-AlfpCre sections. Immunoblotting, of (B) BSEP protein and (C) ABCG8 protein on total membrane

protein extracts from Vps33b$^{fl/fl}$-AlfpCre and Vps33b$^{fl/fl}$ liver, confirms that apical proteins are mislocalised as opposed to down-regulated.

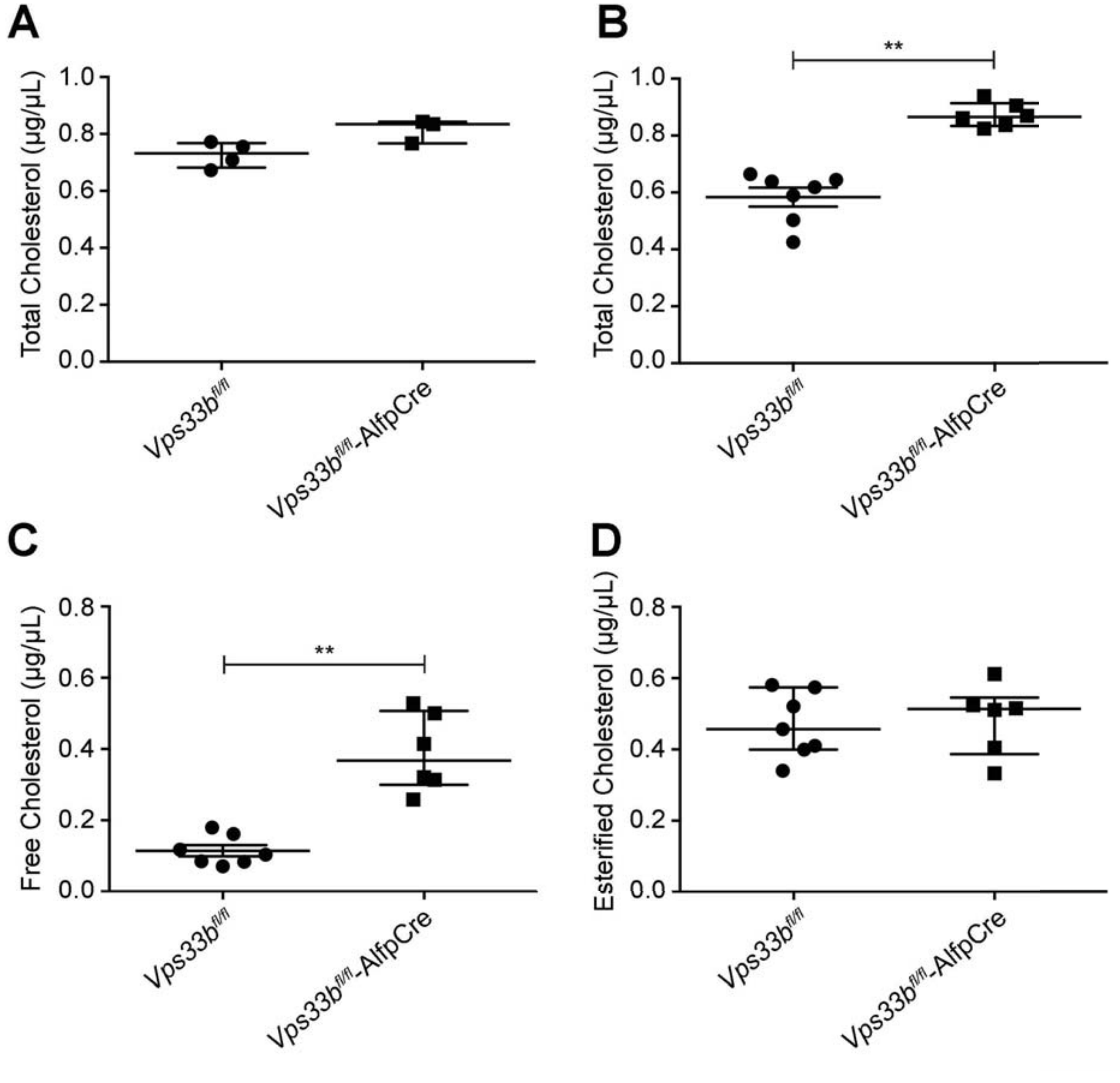

Supplementary Fig.6. Analysis of plasma cholesterol composition. (A) Increase in plasma total cholesterol levels of 14-week-old Vps33b$^{fl/fl}$-AlfpCre mice over Vps33b$^{fl/fl}$ animals was confirmed under normal diet conditions by fluorometry (n=3-4, P=0.1143). Plasma concentrations of (B) total cholesterol (P=0.0012), (C) free cholesterol (P=0.0012) and (D) esterified cholesterol (0.9452) were determined in samples from 14-week-old CA fed Vps33b$^{fl/fl}$-AlfpCre and Vps33b$^{fl/fl}$ mice by fluorometry (n=6-7). All P-values were calculated using the Mann Whitney U test.

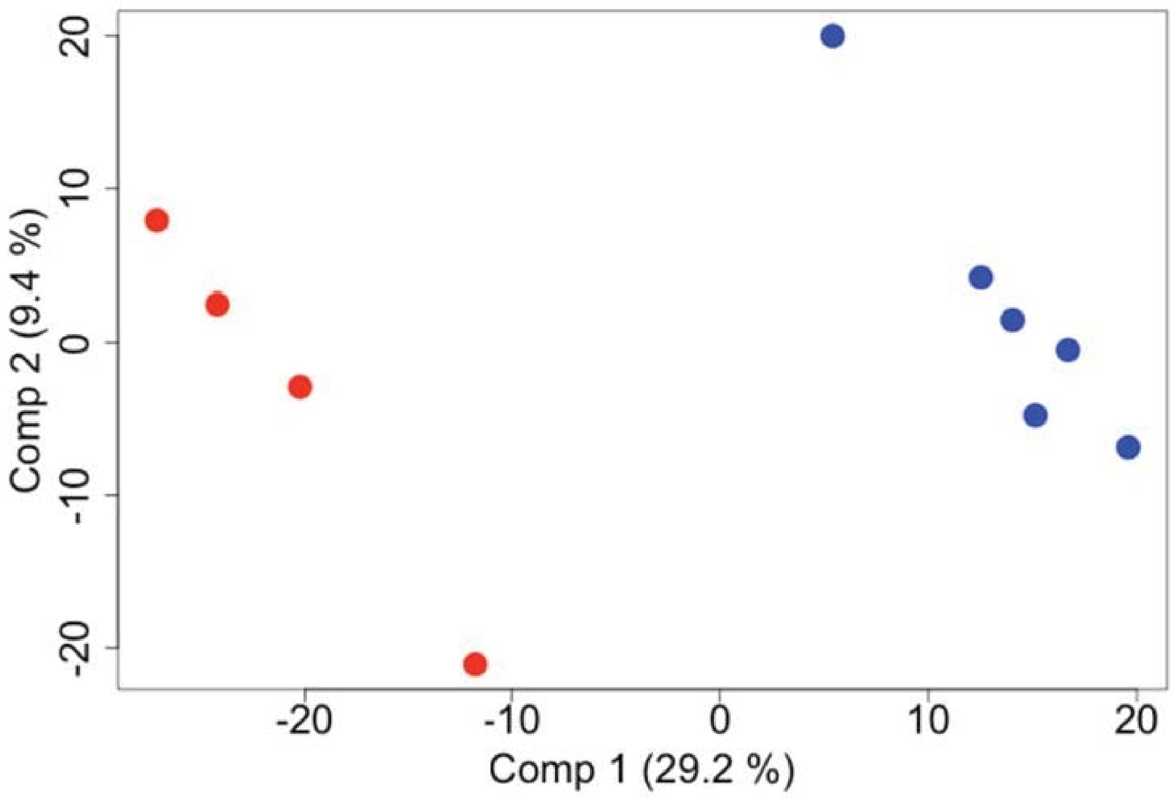

Supplementary Fig.7. PLS scores. PLS scores for 10-14-week-old, cholic acid fed *Vps33b*$^{fl/fl}$ (red) and *Vps33b*$^{fl/fl}$*-AlfpCre* (blue) samples projected onto the first and second components. The first component clearly separates samples from *Vps33b*$^{fl/fl}$ and *Vps33b*$^{fl/fl}$*-AlfpCre* animals.

Sublist	Category	Term	RT	Genes	Count	%	P-Value	Benjamini
	SP_PIR_KEYWORDS	signal	RT		44	32.1	4.6E-7	9.7E-5
	GOTERM_BP_FAT	response to wounding	RT		14	10.2	9.4E-7	9.5E-4
	SP_PIR_KEYWORDS	inflammatory response	RT		8	5.8	1.0E-6	1.1E-4
	UP_SEQ_FEATURE	signal peptide	RT		44	32.1	1.8E-6	7.9E-4
	SP_PIR_KEYWORDS	disulfide bond	RT		38	27.7	1.9E-6	1.3E-4
	GOTERM_BP_FAT	defense response	RT		15	10.9	3.0E-6	1.5E-3
	GOTERM_BP_FAT	inflammatory response	RT		11	8.0	4.1E-6	1.4E-3
	UP_SEQ_FEATURE	metal ion-binding site:Iron (heme axial ligand)	RT		8	5.8	7.5E-6	1.6E-3
	SP_PIR_KEYWORDS	Secreted	RT		26	19.0	9.3E-6	4.9E-4
	SP_PIR_KEYWORDS	glycoprotein	RT		46	33.6	1.3E-5	5.6E-4
	GOTERM_CC_FAT	extracellular region	RT		30	21.9	1.8E-5	2.4E-3
	KEGG_PATHWAY	Retinol metabolism	RT		7	5.1	2.3E-5	1.8E-3
	SP_PIR_KEYWORDS	membrane	RT		60	43.8	3.7E-5	1.3E-3
	UP_SEQ_FEATURE	disulfide bond	RT		35	25.5	4.9E-5	7.0E-3
	SP_PIR_KEYWORDS	heme	RT		8	5.8	5.4E-5	1.6E-3
	SP_PIR_KEYWORDS	microsome	RT		7	5.1	6.4E-5	1.7E-3
	GOTERM_CC_FAT	microsome	RT		9	6.6	6.5E-5	4.4E-3
	GOTERM_CC_FAT	vesicular fraction	RT		9	6.6	8.3E-5	3.7E-3
	UP_SEQ_FEATURE	glycosylation site:N-linked (GlcNAc...)	RT		44	32.1	8.8E-5	9.5E-3
	SP_PIR_KEYWORDS	oxidoreductase	RT		14	10.2	1.5E-4	3.4E-3
	GOTERM_BP_FAT	lipid biosynthetic process	RT		10	7.3	1.8E-4	4.5E-2
	SP_PIR_KEYWORDS	inflammation	RT		4	2.9	2.0E-4	4.1E-3
	UP_SEQ_FEATURE	short sequence motif:Cell attachment site	RT		6	4.4	2.2E-4	1.9E-2
	GOTERM_MF_FAT	electron carrier activity	RT		8	5.8	2.3E-4	5.0E-2
	GOTERM_MF_FAT	heme binding	RT		7	5.1	2.4E-4	2.7E-2
	GOTERM_MF_FAT	tetrapyrrole binding	RT		7	5.1	3.1E-4	2.3E-2
	GOTERM_CC_FAT	cell fraction	RT		14	10.2	6.7E-4	2.3E-2
	GOTERM_CC_FAT	endoplasmic reticulum	RT		17	12.4	6.7E-4	1.8E-2

Supplementary Fig.8. GO annotations. Statistically significant, over-represented GO annotations for the top 150 genes differentially expressed in *Vps33b*$^{fl/fl}$ and *Vps33b*$^{fl/fl}$*-AlfpCre* liver, as identified by DAVID.

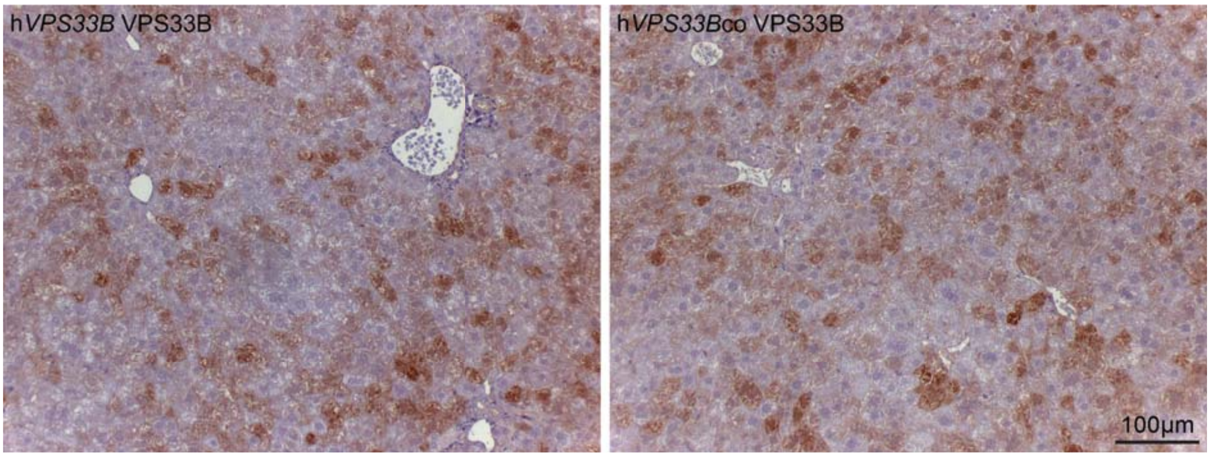

Supplementary Fig.9. Expression of VPS33B following gene transfer. Expression of VPS33B in liver of *Vps33b*$^{fl/fl}$*-AlfpCre* mice injected with ssAAV2/8-EFS-*hVPS33B*-WPRE or ssAAV2/8-EFS-*hVPS33Bco*-WPRE vector was confirmed by immunostaining (n=3). The primary antibody detects human protein only.

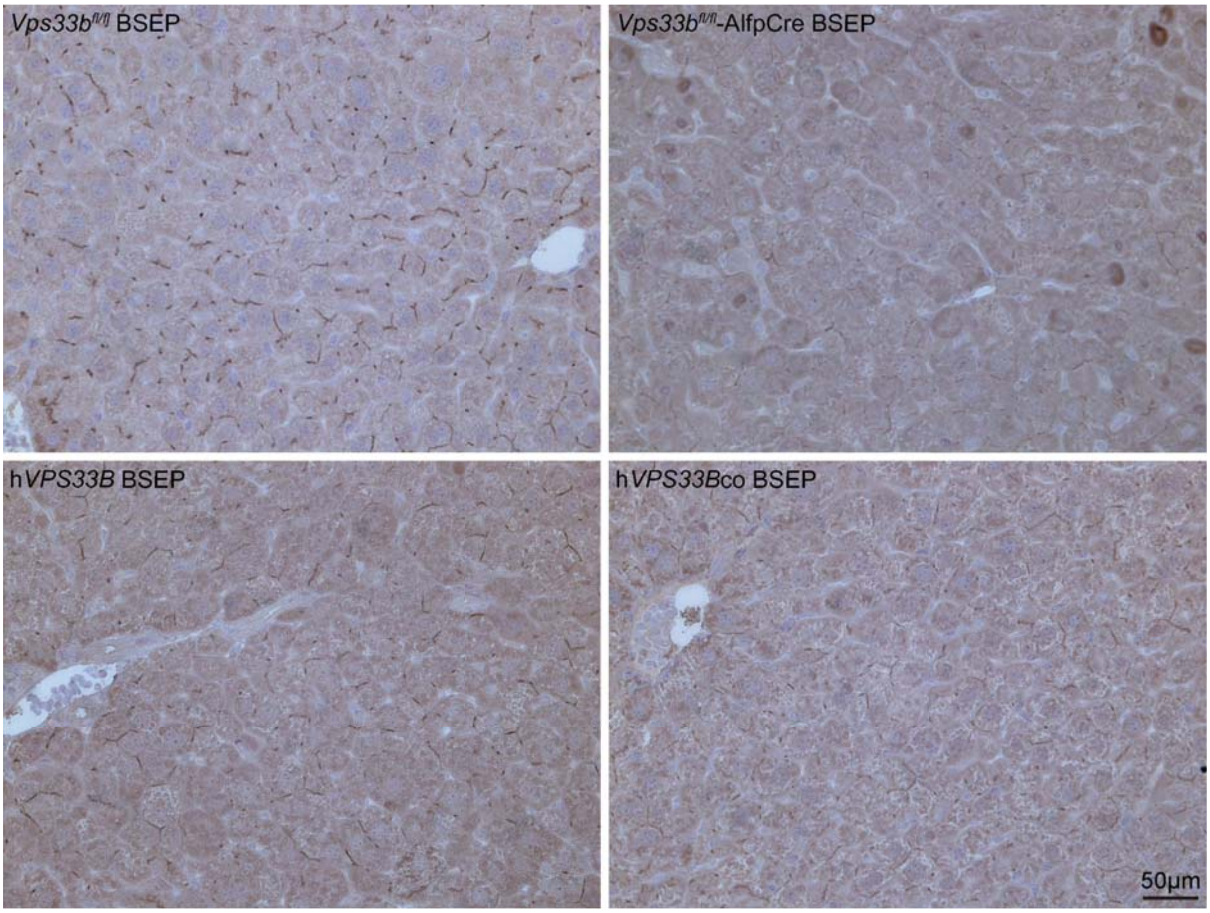

Supplementary Fig.10. Gene transfer restores apical BSEP localisation. Localisation of BSEP protein assessed by immunostaining on *Vps33b$^{fl/fl}$-AlfpCre* and *Vps33b$^{fl/fl}$* liver sections and on sections from *Vps33b$^{fl/fl}$-AlfpCre* mice 9 weeks after injection of ssAAV2/8-EFS-*hVPS33B*-WPRE or ssAAV2/8-EFS-*hVPS33Bco*-WPRE vector (1×10^{12}vg/mouse injected at 5 weeks of age) (n=3). All mice were fed a cholic acid supplemented diet and sacrificed at 14-15 weeks of age.

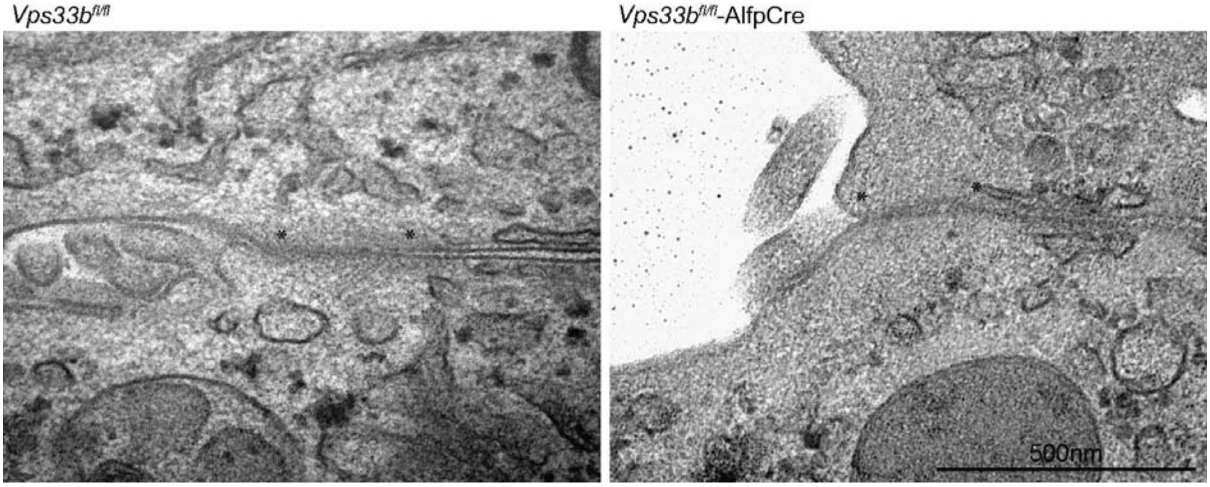

Supplementary Fig.11. Tight junction ultrastructure. Transmission electron micrographs of tight junctions in Vps33b$^{fl/fl}$ and Vps33b$^{fl/fl}$-AlfpCre mouse liver. Tight junction length was measured using ITEM. All mice were fed a normal chow diet and sacrificed at 14-15 weeks of age. Asterisks mark the ends of the tight junction.

Supplementary Tables

Supplementary Table 1. Pathways disrupted by loss of Vps33b expression in liver. Top 10 pathways perturbed in *Vps33b$^{fl/fl}$-ALFPCre* mouse liver, identified by GSEA and ranked by p-value.

Pathway	Pathway score	% of genes measured	P-value	Adjusted P-value
KEGG PRIMARY BILE ACID BIOSYNTHESIS	2.99	93.75	1.00E-04	0.0062
KEGG PPAR SIGNALING PATHWAY	0.96	91.30	1.00E-04	0.0062
KEGG CYTOKINE-CYTOKINE RECEPTOR INTERACTION	0.56	83.52	1.00E-04	0.0062
KEGG ARACHIDONIC ACID METABOLISM	0.97	77.59	5.00E-04	0.0186
KEGG TOLL LIKE RECEPTOR SIGNALING PATHWAY	0.69	95.10	5.00E-04	0.0186
KEGG MAPK SIGNALING PATHWAY	0.43	97.00	8.00E-04	0.0248
KEGG CHEMOKINE SIGNALING PATHWAY	0.47	89.47	0.001	0.0266
KEGG DRUG METABOLISM CYTOCHROME P450	0.98	51.39	0.0014	0.0289
KEGG PATHOGENIC ESCHERICHIA COLI INFECTION	0.88	83.05	0.0014	0.0289
KEGG STEROID HORMONE BIOSYNTHESIS	1.05	54.54	0.002	0.0372

Supplementary Table 2. Antibody list. Details of antibodies used for immunostaining.

Antigen	Host Species	Source	Antigen retrieval	Dilution	Controls
ABCG8	Rabbit	A gift from Prof. Bruno Stieger	95°C, 40min in a pressure cooker. Tris-EDTA buffer pH9	1:100	Negative control (staining mouse liver with secondary antibody only)
BSEP	Guinea pig	Custom made by Eurogentec (Southampton, UK). Peptide sequence: C+SDGSHNNDKKSRLQD-nh2	95°C, 40min in a pressure cooker. Tris-EDTA buffer pH9	1:200	Negative control (staining mouse liver with secondary antibody only)
CC3	Rabbit	Catalogue number: 9961 (Cell Signalling Technology, Leiden, Netherlands)	Microwaved on full power 10min. Warm citrate buffer pH6	1:300	Negative control (staining mouse liver with secondary antibody only)
CEA	Rabbit	Catalogue number: A0115 (DAKO, Ely, UK)	95°C, 40min in a pressure cooker. Tris-EDTA buffer pH9	1:200	Negative control (staining mouse liver with secondary antibody only)
CLDN 1	Rabbit	Catalogue number: 717800 (Thermo Fisher Scientific, Loughborough, UK)	95°C, 40min in a pressure cooker. Tris-EDTA buffer pH9	1:200	Negative control (staining mouse liver with secondary antibody only)
OATP1B2	Rabbit	A gift from Prof. Bruno Stieger	95°C, 40min in a pressure cooker. Tris-EDTA buffer pH9	1:100	Negative control (staining mouse liver with secondary antibody only)
MRP2	Rabbit	A gift from Prof. Bruno Stieger	95°C, 40min in a pressure cooker. Tris-EDTA buffer pH9	1:200	Negative control (staining mouse liver with secondary antibody only)
VPS33B	Rabbit	Catalogue number: 12195-1-AP (Proteintech, Manchester, UK)	95°C, 40min in a pressure cooker. Tris-EDTA buffer pH9	1:100	Negative control (staining mouse liver with secondary antibody only)

References

1. Mazzacuva F, Mills P, Mills K, Camuzeaux S, Gissen P, Nicoli ER, Wassif C, et al. Identification of novel bile acids as biomarkers for the early diagnosis of Niemann-Pick C disease. FEBS Lett 2016; 11:1651-62
2. Wold S, Ruhe A, Wold H, Dunn WJ. The collinearity problem in linear-regression - the partial least-squares (Pls) approach to generalized inverses. SIAM J Sci and Stat Comput 1984;5:735-743.
3. Mevik BH, Wehrens R. The pls package: Principal component and partial least squares regression in R. J Stat Softw 2007;18:1-23.
4. Huang da W, Sherman BT, Lempicki RA. Systematic and integrative analysis of large gene lists using DAVID bioinformatics resources. Nat Protoc 2009;4:44-57.
